# Effects of Humic Substance Amendments on Cadmium and Arsenic Availability, Concentrations, and Potential Health Risk in Rice

**DOI:** 10.3390/toxics14100842

**Published:** 2026-09-22

**Authors:** Bo Li, Mengke Han, Yuyan Chang, Chao Xu, Jie Zhang, Taowen Pan, Zeli Li, Jibiao Geng

**Affiliations:** 1 College of Resources and Environment, Linyi University, Key Laboratory of Green Functional Fertilizer Innovation, Ministry of Agriculture and Rural Affairs, Linyi 276000, China; 13103300915@163.com (M.H.);; 2 Jinzhengda Ecological Engineering Group Co., Ltd., Linyi 276000, China; 3 Institute of Subtropical Agriculture, Chinese Academy of Sciences, Changsha 410125, China

**Keywords:** cadmium, arsenic, fulvic acid, humic acid, paddy soil

## Abstract

Cadmium (Cd) and arsenic (As) co-contamination in paddy soils poses a major challenge for rice safety. Humic substances have the potential to regulate the mobility of these metal(loid)s, yet the distinct effects of different humic substances on Cd/As concentrations, distribution in rice and dietary health risks remain unclear. Hence, a pot experiment was conducted using a historically mining-impacted Cd/As co-contaminated paddy soil to compare the effects of two commercial amendments, fulvic acid (FA) and humic acid (HA), under carbon-equivalent application rates on Cd/As extractability and concentration-based distribution across the soil–root surface-grain continuum and the associated potential dietary risk. Each treatment consisted of three replicate pots, with each pot serving as the experimental unit (39 pots in total). The results showed that adding FA and HA decreased CaCl_2_-Cd by 24.64–67.63%, while increasing the KH_2_PO_4_-As by 15.83–44.55%. This reduction in Cd extractability was accompanied by an increase in Fe/Mn oxide-bound Cd. Moreover, the increase in extractable As was associated with increases in soluble and Al-bound As, and a decrease in Fe-bound As. These changes were associated with increased organic carbon, lower Eh, and changes in Fe oxide fractions. The FA and HA additions also decreased grain Cd concentrations to 0.05–0.30 mg kg^−1^, corresponding to reductions of 30.95–87.68%. However, grain As concentrations showed amendment- and dose-dependent responses, ranging from 0.48 to 0.62 mg kg^−1^ and decreasing by 10.50–12.47% at higher FA rates but increasing by 12.09–14.21% under some HA treatments. These contrasting grain As responses were associated with changes in plaque-associated As and concentration-based transfer factors, suggesting altered As distribution among rice tissues. Correlation and Random Forest analyses indicated that DOC, metals in iron plaque, and pH were the variables most strongly associated with grain Cd/As variation in this study. Because inorganic As was not determined, total grain As was used as a conservative surrogate for inorganic As in the screening-level health risk assessment. Under this assumption, HQ-As was the dominant contributor to HI, and all HI values remained greater than 1, despite reductions in HQ-Cd. Overall, both amendments reduced grain Cd concentration, but their combined Cd/As responses varied across application rates. Higher FA rates reduced both grain Cd and grain As, whereas intermediate HA rates increased grain As despite reductions in grain Cd. These findings demonstrate amendment- and dose-dependent Cd/As trade-offs across the soil–rice continuum and highlight that increased soil As extractability does not necessarily translate into increased grain As concentration.

## 1. Introduction

Cadmium (Cd) and arsenic (As) contamination in paddy soils has raised widespread environmental and public health concerns [1,2]. Toxic metals have caused severe contamination of paddy soils, which may contain either single or co-contaminants of Cd/As [2,3]. Rice (Oryza sativa L.), a staple crop globally, readily accumulates Cd/As through root uptake, which are subsequently transported to edible grains [4,5]. Long-term dietary intake of Cd/As via rice consumption is linked to various adverse health effects, including renal, skeletal, cardiovascular, and carcinogenic risks [3]. Therefore, reducing grain Cd/As accumulation is essential for both soil remediation and food safety protection.

Simultaneous control of soil Cd/As mobility is challenging due to their tendency to respond differently to changes in soil conditions [6,7]. Cd mobility in soil is strongly influenced by adsorption and precipitation processes, which are regulated by soil pH and redox potential [8]. In contrast, the availability of As is closely linked to Fe oxide dynamics and redox-driven transformations [7]. Previous studies have indicated that lower redox potential may reduce Cd mobility through precipitation or redistribution into less labile fractions under flooded conditions, but it may increase As mobility through the reductive dissolution of As-bearing Fe oxides [9,10]. However, these contrasting responses are not controlled by redox potential alone; sulfur-related precipitation, Fe redox cycling, As redox transformation, organic matter decomposition, and microbial activity may also interact to regulate Cd immobilization and As mobilization in flooded conditions [7,9,10]. Such contrasting behavior may result in a trade-off between Cd and As in paddy soils, where an amendment that immobilizes Cd could simultaneously mobilize As.

Humic substances are major components of soil organic matter, and they have been widely used or proposed as soil amendments to regulate metal(loid) mobility and bioavailability [11,12]. Among the humic substance amendments, fulvic acid and humic acid are two commonly used materials for regulating metal(loid) mobility. These amendments may differ in molecular composition, solubility, pH, feedstock origin, production process, and associated ion inputs, which may jointly contribute to their distinct effects on Cd and As behavior [7,13,14]. Previous studies demonstrated that humic substances can immobilize Cd by complexing with organic molecules, regulating pH, and altering the Cd speciation [15]. However, humic substances might also enhance As mobility by competing for adsorption sites, changing Fe oxide dynamics, and affecting As reduction [14]. Moreover, dissolved organic carbon (DOC) derived from humic substances may further influence Cd and As mobility by forming soluble metal–organic complexes, competing with oxyanions for adsorption sites, and altering Fe redox cycling [7,14]. These DOC-mediated processes may affect soil metal(loid) availability, root exposure, and Cd/As transfer in the soil–rice system [8,11,15]. In addition, changes in soil Cd/As availability do not necessarily translate directly into grain accumulation, because Cd and As transfer to rice grains involves distinct processes of soil-to-root uptake, root-to-shoot translocation, and grain loading [16]. From a toxicological viewpoint, the amendment performance should be evaluated not only by soil Cd/As availability or grain Cd/As reduction, but also by dietary health risk [3,17]. For As, this evaluation should distinguish between total As and inorganic As because As toxicity and dietary risk are strongly species-dependent. Therefore, the effects of humic substance amendments on Cd/As co-contaminated paddy soils need to be evaluated from both remediation and health-risk perspectives.

Iron oxides in soil and iron plaque on root surfaces are key interfaces that regulate the Cd/As transfer in flooded rice systems [18,19]. Soil Fe oxides can immobilize Cd/As through adsorption or co-precipitation, while their redox cycling may release the associated metal(loid)s into soil solution under reducing conditions [20]. Iron plaque on rice root surfaces may act either as a barrier to limit Cd/As uptake, or serve as a temporary reservoir that releases Cd/As under changing rhizosphere conditions [19]. Humic substances may regulate the retention and transfer of Cd/As by affecting Fe oxide dynamics, thereby influencing the iron plaque formation [15]. However, the effects of humic substances from different sources on Cd/As behavior at the soil–root interface remain insufficiently understood.

Overall, although humic substances have been widely studied for regulating Cd/As mobility in paddy soils, their effects are often evaluated using soil extractability or grain concentrations separately, and direct comparisons among humic amendments are frequently confounded by differences in application rates and carbon inputs. It therefore remains unclear whether different humic amendments produce comparable Cd/As trade-offs when applied on a carbon-equivalent basis, and whether amendment-induced changes in soil Cd/As extractability are consistently translated into root-surface retention, tissue distribution, and final grain concentrations. To address this gap, a pot experiment was conducted using Cd/As co-contaminated paddy soil amended with fulvic acid (FA) and humic acid (HA) under carbon-equivalent application rates. Specifically, this study aimed to: (1) compare the effects of FA and HA on Cd/As extractability and fractionation in soil; (2) clarify differences and key processes involved in FA- and HA-regulated Cd/As concentrations and distribution in rice; and (3) evaluate the potential health risk of Cd/As exposure via rice consumption.

## 2. Materials and Methods

### 2.1. Test Soil and Amendments

The test soil (0–20 cm) was collected from a paddy field in Liuyang, Hunan Province (28°29′04″ N, 113°37′12″ E), which was contaminated with Cd/As. The contamination of the field was mainly due to historical mining in the nearby Qibaoshan mining area. The sampled soil was air-dried, and large stones and plant roots were removed. The soil was then passed through a 10 mm sieve and mixed thoroughly. The test soil had a pH of 6.03 and it had 19.86 g kg^−1^ soil organic carbon (SOC), 2.07 mg kg^−1^ total Cd, and 147.06 mg kg^−1^ total As, with 11.80% clay, 75.45% silt, and 12.75% sand.

The fulvic acid amendment (FA) was sourced from Shandong New Tranlin Holdings Co., Ltd. (Shandong, China). It was made from wheat straw by ammonium sulfite ((NH_4_)_2_SO_3_) digestion at 160 °C and 0.2 MPa for 3 h, followed by concentration. The FA had a pH of 5.43, a total organic carbon (TOC) concentration of 28.24%, a total Cd concentration of 0.04 mg kg^−1^, and a total As concentration of 0.02 mg kg^−1^. The humic acid amendment (HA) was obtained from Shandong Chuangxin Humic Acid Technology Co., Ltd. (Liaocheng, China). It was a commercial sodium humate product derived from peat and produced by alkaline extraction using NaOH solution, followed by drying to get solid sodium humate. The HA had a pH of 10.27, a TOC concentration of 30.54%, a total Cd concentration of 0.03 mg kg^−1^, and a total As concentration of 0.11 mg kg^−1^. The amendments were characterized for pH, TOC, total Cd, and total As, whereas total N, total S, Na, and other accompanying constituents were not determined in this study.

### 2.2. Pot Experiment

A pot experiment was conducted using two humic substances (FA and HA) and seven application rates (0, 0.5, 1, 2, 3, 4, and 5 g C kg^−1^ soil) in a greenhouse under natural photoperiod and ambient temperature conditions. Treatments were designated as follows: CK, no amendment; FA1 and HA1, 0.5 g C kg^−1^ soil; FA2 and HA2, 1 g C kg^−1^ soil; FA3 and HA3, 2 g C kg^−1^ soil; FA4 and HA4, 3 g C kg^−1^ soil; FA5 and HA5, 4 g C kg^−1^ soil; FA6 and HA6, 5 g C kg^−1^ soil. Because FA and HA had different TOC concentrations, the same carbon-equivalent doses corresponded to different actual amendment masses. The corresponding actual application rates are shown in Table 1. The amendment rates were selected on a carbon-equivalent basis to compare FA and HA under equivalent organic carbon inputs and to evaluate dose-response patterns. The higher rates corresponded to relatively large actual amendment masses, and this pot experiment was therefore designed as a controlled comparative dose-response study rather than as a direct field application recommendation. Each treatment had three replicates, totaling 39 pots.

The homogenized soil was thoroughly mixed with the appropriate amount of FA or HA and then transferred into plastic pots (20 cm high and 25 cm in diameter), with 5 kg of soil per pot. Before rice transplanting, basal fertilizers were applied to each pot at 1.95 g urea, 0.8 g NH_4_H_2_PO_4_, and 1.7 g KCl. The rice cultivar used in this experiment was Zhuliangyou 189, a two-line hybrid indica rice from Hunan Yahua Seed Industry Co., Ltd. (Changsha, China). After 7 days of flooding, 30-day-old rice seedlings with uniform growth were transplanted on 19 April 2019, with three plants maintained per pot, and were harvested at maturity on 24 July 2019, after 96 days of cultivation. After transplanting, all pots were maintained under flooded conditions throughout the rice growth period, with a standing water layer of approximately 3–5 cm above the soil surface. Deionized water was added daily to maintain the target water depth. No intentional drainage was performed before harvest. The experiment was conducted using a completely randomized design, with three replicate pots per treatment. Pot positions were periodically re-randomized during the experiment to minimize potential positional effects within the greenhouse.

### 2.3. Sample Collection and Analysis

At the maturity stage of rice, soil redox potential (Eh) was measured in situ immediately before sampling at ambient temperature using a platinum electrode with an Ag/AgCl reference electrode (FJA, Chuan-Di Instrument & Equipment Co., Ltd., Nanjing, China). Readings were recorded after the instrument automatically indicated signal stabilization. For each pot, three stabilized readings from different positions were averaged for analysis. Eh values were reported relative to the Ag/AgCl reference electrode without correction to the standard hydrogen electrode. Paired soil and rice plant samples were then collected. Rice plants were thoroughly rinsed with ultrapure water. Fresh root subsamples were immediately placed in sealed polyethylene bags, and then kept for further determination. The remaining plant materials were processed by heating them at 105 °C for 30 min, and then dried at 70 °C until a constant weight was achieved. After that, the plants were divided into roots, stems, leaves, and grains (brown rice). The corresponding tissues from the three rice plants within each pot were pooled to form one composite sample for each tissue type. Each composite tissue sample was ground finely by a stainless-steel grinder (FW-80, Beijing Ever Light Medical Equipment Co., Ltd., Beijing, China), sealed in polyethylene bags, and stored before analysis. Soil samples were collected, and then split into two parts after homogenization. The fresh portion was analyzed immediately for soil pH, CaCl_2_-extractable Cd, and KH_2_PO_4_-extractable As, whereas the second portion was freeze-dried at −70 °C and was reserved for metal fractionation analysis.

Soil pH, total Cd/As, CaCl_2_-extractable Cd, and KH_2_PO_4_-extractable As were measured following the methods reported by Li [14], with minor modifications. Soil Cd/As fractions were extracted using the modified sequential extraction procedures described by [7]. These sequentially extracted fractions were operationally defined and should not be interpreted as discrete chemical species. Treatment comparisons and treatment-related changes were based on the absolute concentrations of individual operational fractions rather than on percentage distributions, because percentage changes may reflect relative shifts among fractions rather than true changes in fraction mass. The TOC and dissolved organic carbon (DOC) were determined following Zhang [21]. Dithionite–citrate–bicarbonate-extractable Fe (Fed) and acid ammonium oxalate-extractable Fe (Feo) were extracted and quantified as operationally defined Fe fractions according to Li [22]. Fe concentrations in the extracts were determined by ICP-OES (5110, Agilent Technologies, Santa Clara, CA, USA). The metals (Cd, As, Fe, Mn) in iron plaque (Cd_IP_, As_IP_, Fe_IP_, and Mn_IP_) and Cd/As concentrations in different rice tissues were determined following Li [23]. Specifically, iron plaque was extracted using the DCB method as described in Method SIV. Approximately 2.0 g of fresh roots rinsed with deionized water was immersed in 40 mL of citrate–bicarbonate extracting solution for 10 min, followed by the addition of 1.0 g sodium dithionite and extraction for another 50 min. The extract was transferred to a 100 mL volumetric flask and diluted to volume with ultrapure water. Plaque-associated Fe, Mn, Cd, and As were determined in the DCB extracts and normalized to the dry weight of the corresponding root. After DCB extraction, the roots were rinsed with deionized water, dried, ground, and used for root tissue Cd/As analysis. A detailed description of the analytical procedures is provided in the Supporting Information (SI–SV).

Quality assurance and quality control (QA/QC) procedures were applied during sample digestion, extraction, and instrumental analysis. Procedural blanks were included during soil digestion/extraction, DCB extraction, and plant digestion procedures. External calibration was performed using certified standard solutions, with calibration coefficients greater than 0.999. Calibration verification standards were analyzed periodically during instrumental analysis. Certified reference materials were used to evaluate analytical accuracy. For soil Cd and As analyses, reference soil GBW-070011 was used, with recoveries of 87.2–113.5% for Cd and 90.1–107.7% for As. For sequential extraction, the recoveries of Cd and As were 92.3–105.2% and 89.7–112.4%, respectively. For rice tissue analysis, bush twigs and leaves (GBW-07602) were used, with Cd and As recoveries of 96.4–105.3% and 92.1–99.5%, respectively. Instrumental performance parameters for the ICP-MS (7900, Agilent Technologies, Santa Clara, CA, USA) and ICP-OES (5110, Agilent Technologies, Santa Clara, CA, USA) used in this study are summarized in Table S2.

### 2.4. Calculation and Statistical Analysis

The transfer factors (*TF*s) of Cd/As in rice plants were calculated as concentration-based ratios to describe their relative distribution among roots, stems, and grains. The *TF*_root-stem_ and *TF*_stem-grain_ were calculated as follows:

*TF*_root-stem_ = *C*_stem_/*C*_root_ (1)

*TF*_stem-grain_ = *C*_grain_/*C*_stem_ (2)

where *C*_root_, *C*_stem_, and *C*_grain_ represent Cd or As concentrations in roots, stems, and grains, respectively. These *TF*s were used as relative concentration ratios rather than direct measures of elemental flux or physiological translocation.

The potential health risks of Cd/As exposure through rice consumption were evaluated using the estimated daily intake (*EDI*), hazard quotient (*HQ*), and hazard index (*HI*). The *EDI* was calculated as follows:

*EDI* = *C*_grain_ × *IR*/*BW* (3)

where *IR* is the daily rice ingestion rate and *BW* is the average body weight. In this study, a rural adult exposure scenario was used. The *IR* was set as 0.389 kg day^−1^ on an uncooked-rice-equivalent basis [24], and the *BW* was set as 60 kg to represent an adult body weight commonly used in dietary exposure assessment for Chinese rural populations [3]. Cd and As concentrations in rice grains were determined on a dry-weight basis, and the exposure calculation was therefore performed using the same uncooked/dry grain basis without cooked-rice moisture conversion. Because inorganic As was not determined in this study, total As concentration in rice grains was used as a conservative surrogate for inorganic As to calculate *HQ*-As and *HI*. This approach represents a conservative screening-level assessment and may overestimate As-related risk because the oral reference dose used for As is based on inorganic As. The *HQ* was calculated as

*HQ* = *EDI*/*HGV* (4)

where *HGV* is the oral health-guidance value used for screening. The *HGV*s used for Cd and As were 1.0 × 10^−3^ (food-based oral reference dose, RfD; U.S. EPA) and 3.0 × 10^−4^ mg kg^−1^ day^−1^ (chronic oral minimal risk level, MRL; ATSDR), respectively [25,26]. The *HI* was calculated as the sum of *HQ* values for Cd/As, and an *HI* value greater than 1 was considered to indicate a potential non-carcinogenic health risk under this conservative screening-level assumption:

*HI* = *HQ*-Cd + *HQ*-As (5)

*HQ* and *HI* are dimensionless screening ratios and should not be interpreted as probabilities of adverse health effects. This assessment used a deterministic approach based on point estimates for rice ingestion rate, body weight, grain Cd and As concentrations, and the total-As surrogate assumption; variability and uncertainty in these parameters were not propagated. Carcinogenic risk indicators, such as incremental lifetime cancer risk (ILCR) or lifetime cancer risk (LCR), were not calculated because inorganic As was not determined, and using total grain As for carcinogenic-risk estimation would introduce additional uncertainty.

Statistical analyses were performed using SPSS Statistics 20.0 (IBM Corp., Armonk, NY, USA). All data are expressed as means ± standard error. The effects of amendment type, carbon-equivalent dose, and their interaction were evaluated using two-factor ANOVA for the nonzero FA and HA treatments. Treatment differences relative to the shared CK were assessed using Dunnett’s test. FA and HA were also compared directly at each equivalent carbon dose, with Bonferroni adjustment for multiple comparisons. The normality of model residuals and homogeneity of variance were assessed using the Shapiro–Wilk and Levene’s tests, respectively, and no data transformation was applied. For selected variables, dose-response relationships were further evaluated separately for FA and HA using linear and quadratic regression models, with regression coefficients, 95% confidence intervals, R^2^, and *p* values reported. Statistical significance was set at *p* < 0.05. Pearson correlation analysis was used to evaluate the relationships among soil properties, Cd/As availability, Cd/As fractions, metals in iron plaque, and Cd/As in rice tissues. Random Forest analysis (RF) was performed in R (4.4.5) as an exploratory approach to rank variables associated with grain Cd and As concentrations using 39 individual pot-level observations and 10 predictors for each model. The models were fitted using 1000 trees. The mtry values were 3 and 9 for the grain Cd and grain As models, respectively, and the node size was set to 1. A fixed random seed was used, and variable importance was evaluated using the mean decrease in accuracy. Model performance was evaluated using out-of-bag (OOB) validation and leave-one-treatment-out grouped validation. Because of the limited sample size, RF results were interpreted as exploratory variable-ranking results rather than validated predictive models. Linear regression analysis was further used to explore the relationships between selected variables and grain Cd/As.

## 3. Results

### 3.1. Soil pH, Eh, Organic Carbon, and Fe Oxide Fractions

Soil pH and redox potential (Eh) were strongly affected by the two humic substance amendments. Soil pH exhibited opposite trends under FA and HA treatments. Compared with CK, soil pH decreased by 0.43 units under FA6 (*p* < 0.05, Figure 1a), whereas no significant decreases were observed under FA1-FA5 treatments. In contrast, soil pH significantly increased by 0.12–0.59 units under HA, with significant increases observed under HA3-HA6 (*p* < 0.05, Figure 1b). Both FA and HA treatments generally decreased the soil Eh. Compared with CK, soil Eh at maturity-stage sampling declined by 89.83–112.95 mV under FA treatments and 56.35–80.95 mV under HA treatments, with significant decreases observed under FA2, FA5, FA6, and HA5 (*p* < 0.05, Figure 1a,b). Notably, the magnitude of the maturity-stage Eh decrease was relatively consistent across FA treatments, whereas greater variation among application rates was observed under HA treatments. Two-factor analysis further showed a significant amendment-type effect and amendment × dose interaction for soil pH (both *p* < 0.001), whereas Eh was affected by amendment type (*p* = 0.017) but showed no significant dose or interaction effects (Table S5).

The total organic carbon (TOC) and dissolved organic carbon (DOC) were enhanced by FA and HA treatments, although their response patterns differed between the two amendments. Compared with CK, TOC remained unchanged under FA1–FA3, but significantly increased by 12.58–16.81% under FA4–FA6 (*p* < 0.05, Figure 1c). Under HA treatments, TOC increased progressively by 4.44–22.96%, with significant increases observed under HA3–HA6 (*p* < 0.05, Figure 1d). For reference, the carbon-equivalent amendment rates of 0.5–5 g C kg^−1^ soil would correspond to theoretical TOC increases of approximately 2.52–25.17% relative to the initial soil organic carbon concentration of 19.86 g kg^−1^, assuming complete retention of added carbon. Thus, the unchanged TOC under FA1-FA3 suggests that the endpoint TOC response was relatively modest at lower FA rates. Similarly, DOC concentrations increased from 15.85 mg kg^−1^ in CK to 40.92–165.62 mg kg^−1^ under FA treatments and to 22.78–115.20 mg kg^−1^ under HA treatments, corresponding to 2.58–10.45-fold and 1.44–7.27-fold increases, respectively. Significant increases relative to CK were observed under all FA treatments and HA2–HA6 (*p* < 0.05, Figure 1c,d). The concentrations of DCB-extractable Fe (Fed) and oxalate-extractable Fe (Feo) showed contrasting qualitative patterns under FA and HA treatments. Fed generally showed lower values under amendment treatments than under CK, whereas Feo generally showed higher values (Figure 1e,f). Two-factor analysis showed significant amendment-type and dose effects for TOC, significant amendment-type, dose, and interaction effects for DOC, a significant dose effect for Feo, and significant dose and amendment × dose effects for Fed (Table S5).

### 3.2. Soil Cd/As Availability and Fractionation

The two humic substance amendments exerted contrasting effects on soil CaCl_2_-extractable Cd (CaCl_2_-Cd) and KH_2_PO_4_-extractable As (KH_2_PO_4_-As). Compared with CK, CaCl_2_-Cd decreased significantly by 24.64–51.05% under FA treatments and by 25.12–67.63% under HA treatments, with stronger reductions observed at higher application rates. Significant reductions were observed under FA4–FA6 and HA2–HA6 (*p* < 0.05, Figure 2a,b). In contrast, KH_2_PO_4_-As increased by 23.49–44.55% under FA treatments and by 15.83–30.20% under HA treatments, with significant increases observed under FA3–FA6, HA3, and HA6 (*p* < 0.05; Figure 2c,d). For both CaCl_2_-Cd and KH_2_PO_4_-As, significant amendment-type and dose effects were detected, whereas the amendment × dose interactions were not significant (Table S5). Dose-response analysis further showed significant linear decreases in CaCl_2_-Cd and linear increases in KH_2_PO_4_-As with increasing dose under both FA and HA (Table S7).

The soil Cd/As fractionation patterns were significantly modified under FA and HA treatments. Compared with CK, EX-Cd decreased by 7.27–36.03% under FA treatments and by 16.96–68.69% under HA treatments (Figure 2e,f). Significant decreases were observed under FA2–FA6 and all HA treatments. In contrast, the carbonate-bound Cd (CA-Cd) and Fe/Mn-bound Cd (OX-Cd) increased under both amendments. CA-Cd increased by 19.09–42.97% under FA treatments and by 18.86–46.41% under HA treatments, while OX-Cd increased by 16.33–27.89% and 18.22–33.23%, respectively. The increases in CA-Cd were significant under all FA treatments and HA1–HA2, whereas OX-Cd increased significantly under all FA treatments and HA1 and HA3 (Figure 2e,f). Under FA treatments, soluble As (AE-As) increased by 17.93–49.44%, and Al-bound As (Al-As) also increased by 55.44–63.09% (Figure 2g,h). Similarly, AE-As and Al-As increased by 12.01–41.86% and 50.38–68.93% under HA treatments, respectively. The increase in AE-As was significant under all FA treatments but not under HA treatments, whereas Al-As increased significantly under all FA and HA treatments. In contrast, the Fe-As decreased by 19.11–28.96% and 18.95–27.36% under FA and HA treatments, respectively. Significant amendment × dose interactions were detected for EX-Cd, CA-Cd, OX-Cd, Al-As, and Fe-As, whereas no significant interaction was observed for AE-As (Table S5).

### 3.3. Cd, As, Mn, and Fe in Iron Plaque

The metals in iron plaque (Cd_IP_, As_IP_, Mn_IP_, and Fe_IP_) were markedly affected by the two humic substance amendments (Figure 3). Compared with CK, Cd_IP_ decreased by 18.16–24.06% and 22.14–55.17% under FA and HA treatments, respectively, although none of these decreases were significant after Dunnett adjustment (Figure 3a,b). Conversely, As_IP_ showed different responses to the two amendments. Compared with CK, As_IP_ generally decreased under FA treatments, with significant decreases observed under FA2–FA6, whereas under HA treatments, HA1 showed a significant decrease but HA2–HA6 showed significant increases of 12.17–44.72% (*p* < 0.05, Figure 3c,d). Mn_IP_ also decreased significantly under both amendments, with significant reductions observed under FA1–FA6 and HA3–HA6 compared to the CK (Figure 3e,f). Under FA treatments, Fe_IP_ generally decreased, but a significant reduction was observed only under FA6 (*p* < 0.05, Figure 3g). Similarly, Fe_IP_ generally decreased under HA treatments, with significant reductions under HA3–HA6 (*p* < 0.05, Figure 3h). Fe-normalized Cd_IP_ and As_IP_ values were further calculated to account for treatment-induced differences in Fe_IP_ (Figure S2). Cd_IP_/Fe_IP_ showed no significant differences among treatments, whereas As_IP_/Fe_IP_ showed an amendment-dependent response, with a clear increase under HA treatments. Two-factor analysis showed that Cd_IP_ was significantly affected by dose (*p* = 0.031), but not by amendment type or the amendment × dose interaction. In contrast, As_IP_ was significantly affected by amendment type, dose, and their interaction (*p* < 0.001). Significant amendment × dose interactions were also observed for Fe_IP_ and Mn_IP_ (Table S5).

### 3.4. Cd/As Concentrations and Distribution in Rice Tissues

The two humic substance amendments reduced Cd concentrations in rice tissues but showed different effects on As concentrations, especially grain As. The Cd concentrations in rice grains were markedly reduced by both FA and HA treatments (Figure 4a,b). Compared with CK, grain Cd concentrations decreased by 30.95–65.95% under FA treatments and 40.85–87.68% under HA treatments (*p* < 0.05), with the largest reductions observed under FA6 and HA6. Grain Cd concentration was significantly affected by dose and the amendment × dose interaction (*p* < 0.001), whereas the overall amendment-type effect was not significant (*p* = 0.204; Table S5). Dose-specific comparisons showed that grain Cd concentration was significantly lower under FA than HA at 0.5 g C kg^−1^ soil, but significantly lower under HA at 4 and 5 g C kg^−1^ soil; no significant FA–HA differences were detected at 1–3 g C kg^−1^ soil after multiple-testing adjustment (Table S6). Grain Cd also decreased linearly with increasing dose under both FA and HA (both *p* < 0.001; Table S7).

The grain As concentrations showed more complex responses to FA and HA treatments (Figure 4c,d). Under FA treatments, the grain As concentrations generally declined, with significant decreases observed under FA1 and FA3–FA6, and reductions of 10.50–12.47% at higher FA rates. Under HA treatments, the grain As concentrations increased significantly under HA1, whereas HA2–HA6 did not differ significantly from CK, although numerical increases were observed under HA2–HA3 (Figure 4d). For reference, the grain Cd concentrations were compared with the Cd limit for rice (0.2 mg kg^−1^). In contrast, the grain As was determined as total As rather than inorganic As; therefore, it was not directly compared with the inorganic As limit. Grain As concentration was significantly affected by amendment type, dose, and their interaction (*p* ≤ 0.001; Table S5). Dose-specific comparisons showed significantly lower grain As concentrations under FA than HA at 2–5 g C kg^−1^ soil (Table S6). Dose-response analysis further showed a significant linear decrease in grain As with increasing FA dose (*p* < 0.001). Under HA, no significant linear trend was detected (*p* = 0.353); the quadratic term was significant (*p* = 0.029), although the overall quadratic model was marginally non-significant (*p* = 0.057), indicating a curved rate-dependent response (Table S7).

The Cd concentrations in vegetative tissues also generally decreased under FA and HA treatments (Figure 4e,f). Compared with CK, leaf, stem, and root Cd concentrations decreased by 13.68–46.35%, 11.76–75.00%, and 24.61–61.87% under FA treatments, and by 16.87–53.73%, 20.81–86.45%, and 33.33–58.22% under HA treatments (*p* < 0.05, Figure 4e,f), respectively. The As concentrations in vegetative tissues showed tissue-specific responses (Figure 4g,h). Under FA treatments, the leaf As decreased by 23.38–37.31%, the stem As increased by 23.60% under FA4 but decreased by 13.36–39.27% under FA5–FA6, and the root As increased by 47.52–61.24% under FA5–FA6 (*p* < 0.05). Under HA treatments, the leaf As generally decreased by 8.50–25.70%, the stem As remained unchanged under HA1-HA3 but increased by 57.64–76.21% under HA4–HA6, and the root As increased by 17.75–41.80% under HA1 and HA3 but decreased by 25.91% under HA6 (*p* < 0.05). Most tissue Cd/As concentrations showed significant amendment × dose interactions (Table S5).

To further reveal the Cd/As distribution in rice plants, the *TF* values of Cd/As from root to stem (*TF*_root-stem_) and from stem to grain (*TF*_stem-grain_) were calculated (Figure S1). Compared with CK, the *TF*_stem-grain_ of Cd decreased under FA2-FA6 and HA3, HA5, and HA6, whereas the *TF*_stem-grain_ of Cd differed significantly from CK only under FA3. The *TF*_root-stem_ and *TF*_stem-grain_ of As showed different responses to FA and HA treatments (Figure S1e–h). Compared with CK, the *TF*_root-stem_ of As decreased under FA5–FA6 and HA1 but increased under HA4–HA6, whereas the *TF*_stem-grain_ of As increased under FA6 and decreased under HA4–HA6. Significant amendment × dose interactions were observed for all four concentration-based transfer factors (Table S5). The corresponding mean ± SE values for Cd- and As-related variables are provided in Tables S3 and S4, respectively.

### 3.5. Screening-Level Dietary Risk Assessment of Cd/As Exposure Through Rice Consumption

The screening-level dietary risk of Cd and As through rice consumption was assessed using *HQ* and *HI* as screening indices (Figure 5). *HQ*-As was substantially higher than *HQ*-Cd and dominated *HI* across all treatments. Although both amendments reduced *HQ*-Cd, *HI* values remained greater than 1 in all treatments. Compared with CK, *HQ*-Cd decreased by 30.95–65.95% under FA treatments and by 40.85–87.67% under HA treatments. However, *HQ*-As changed only slightly under FA treatments and under HA1 and HA4–HA6, whereas larger increases were observed only under HA2–HA3. Accordingly, the *HI* decreased by 6.57–15.67% under FA2–FA6 and by 8.35–12.55% under HA4–HA6, whereas *HI* increased slightly under HA1–HA3. The largest reductions in *HI* were observed under FA6 and HA5 for FA and HA treatments, respectively. The corresponding EDI, HQ, and HI values for all treatments are provided in Table S1.

### 3.6. Correlations Among Soil Properties, Extractable Cd/As, Iron Plaque, and Cd/As in Rice Tissues

Correlation analysis showed that the relationships among soil properties, extractable Cd/As, metals in iron plaque, and Cd/As in rice tissues differed between FA and HA treatments (Figure 6). Under FA treatments, grain Cd showed positive correlations (Figure 6a, *q* < 0.05) with root Cd, Fe_IP_, Mn_IP_, CaCl_2_-Cd, pH, Eh, and Fed, whereas negative correlations were observed with DOC, SOC, and Feo. CaCl_2_-Cd was positively correlated with Eh and Fed, but negatively correlated with DOC, SOC, and Feo. Cd_IP_ also showed a positive correlation with Fe_IP_. Grain As was positively correlated with As_IP_, Fe_IP_, Mn_IP_, pH, and Fed, but negatively correlated with root As, KH_2_PO_4_-As, DOC, SOC, and Feo. As_IP_ was positively correlated with Mn_IP_, pH, Eh, and Fed, whereas negative correlations were observed with KH_2_PO_4_-As, DOC, SOC, and Feo. KH_2_PO_4_-As also showed positive correlations with SOC, DOC, and Feo, but negative correlations with pH, Eh, and Fed (*q* < 0.05). Under HA treatments, grain Cd showed positive correlations (Figure 6b, *q* < 0.05) with root Cd, Cd_IP_, Fe_IP_, Mn_IP_, CaCl_2_-Cd, and Fed, whereas negative correlations were observed with pH, DOC, SOC, and Feo. CaCl_2_-Cd was positively correlated with Eh and Fed, but negatively correlated with pH, DOC, SOC, and Feo. Cd_IP_ also showed positive correlations with Fe_IP_, Mn_IP_, CaCl_2_-Cd, and Eh, but negative correlations with DOC and SOC. Grain As showed no significant correlations with soil properties, KH_2_PO_4_-As, metals in iron plaque, or As in roots after FDR correction. However, As_IP_ was positively correlated with KH_2_PO_4_-As, pH, DOC, SOC, and Feo, whereas negative correlations were observed with Fe_IP_, Mn_IP_, Eh, and Fed. KH_2_PO_4_-As also showed positive correlations with pH, DOC, SOC, and Feo, but negative correlations with Eh and Fed (*q* < 0.05).

Collectively, soil pH, Eh, DOC, SOC, and Fe oxide fractions showed amendment-specific associations with extractable Cd/As under FA and HA treatments. Extractable Cd/As, metals in iron plaque, and soil properties were also associated with Cd/As in rice grains, although As responses were more amendment-dependent than Cd.

### 3.7. Exploratory Variables Associated with Grain Cd/As Concentrations

Random Forest analysis (RF) was further used as an exploratory approach to rank variables associated with grain Cd/As concentrations within this dataset (Figure 7). For the grain Cd model, the OOB R^2^, OOB MSE, and OOB RMSE were 0.82, 0.0028, and 0.053, respectively. For the grain As model, the OOB R^2^, OOB MSE, and OOB RMSE were 0.69, 0.0014, and 0.037, respectively. Leave-one-treatment-out grouped validation yielded R^2^ and RMSE values of 0.72 and 0.066 for grain Cd, and 0.59 and 0.041 for grain As, respectively.

In the grain Cd model, DOC had the highest importance for grain Cd, followed by Cd_IP_, SOC, CaCl_2_-Cd, and pH (Figure 7a). In the grain As model, DOC also ranked first, followed by As_IP_, SOC, Mn_IP_, and pH (Figure 7b). KH_2_PO_4_-As ranked lower than As_IP_ in the grain As model. Overall, DOC and metals in iron plaque ranked among the variables with relatively high importance in both models.

## 4. Discussion

### 4.1. Effects of Humic Substance Amendments on Soil Cd/As Mobility and Redistribution

The two humic substance amendments decreased CaCl_2_-extractable Cd (CaCl_2_-Cd) but increased KH_2_PO_4_-extractable As (KH_2_PO_4_-As) (Figure 2). Because NH_4_H_2_PO_4_ was applied to all treatments, KH_2_PO_4_-As differences among treatments were interpreted under a comparable P input background, while KH_2_PO_4_-As itself represents an operational phosphate-desorbable As pool rather than direct in situ plant-available As. Correlation analysis showed that CaCl_2_-Cd and KH_2_PO_4_-As were closely associated with increases in organic carbon and changes in soil properties (Figure 6). Humic substances are generally rich in oxygen-containing functional groups, which can alter Cd/As mobility and bioavailability [13,28]. In this study, both SOC and DOC increased with FA and HA addition, although DOC showed greater amendment-specific variation across application rates. Both carbon pools showed opposite relationships with CaCl_2_-Cd and KH_2_PO_4_-As (Figure 6), suggesting that the increase in organic carbon was associated with lower Cd extractability but higher As mobilization in soil. The decrease in Cd mobility may be partly related to interactions between Cd and humic-derived functional groups, as reported in previous studies [22,29]. However, the sequential extraction results showed that Cd was redistributed mainly toward CA-Cd and OX-Cd, rather than showing a dominant increase in OM-Cd fractions (Figure 2). Therefore, organic complexation should be regarded as a possible contributing process, while the present results more directly support Cd redistribution toward CA- and OX-associated fractions. In contrast, the increase in KH_2_PO_4_-As may be related to competition between dissolved organic ligands and As oxyanions for adsorption sites on soil minerals or by facilitating the dissolution/transformation of As-bearing Fe oxides [30]. Similar contrasting changes in Cd/As availability with humic substances and organic amendments have also been reported in contaminated paddy soils [7,11]. Notably, the increase in DOC in soil may also increase Cd mobility by forming soluble Cd-DOC complexes [8]. This may be related to differences in DOC source, molecular weight, aromaticity, and soil conditions [31,32].

Soil Eh declined with both FA and HA and was positively correlated with CaCl_2_-Cd but negatively correlated with KH_2_PO_4_-As (Figure 1 and Figure 6). This indicates that the low endpoint redox conditions under humic substance amendments were also associated with Cd/As availability. Because Eh was measured only at soil sampling, the results should be interpreted as endpoint redox status rather than temporal redox dynamics during rice growth. Lower Eh may facilitate changes in Fe-associated pools, thereby altering the retention and release behavior of Cd/As [14,20]. In this study, Fed decreased whereas Feo increased with both amendments, and both Fe fractions were closely related to CaCl_2_-Cd and KH_2_PO_4_-As (Figure 1 and Figure 6). However, these extraction-based results do not demonstrate mineralogical transformation from one Fe pool to another. Similarly, the concurrent increase in Feo and OX-Cd should be interpreted as indicative of coupled changes in extractable Fe pools and Cd redistribution toward Fe/Mn oxide-associated fractions, rather than as direct proof of Cd adsorption onto newly formed reactive Fe oxides [22]. This pattern was consistent with the decrease in CaCl_2_-Cd. In contrast, the combination of lower Eh, decreased Fed and Fe-As, and increased KH_2_PO_4_-As was consistent with a possible reductive Fe-related As mobilization pathway [33]. Soil pH further strengthened this divergence, especially under HA treatments (Figure 1b). The HA-induced increase in pH may have further reduced Cd solubility by enhancing Cd adsorption and precipitation [34,35], whereas this pH-related explanation was less applicable to FA treatments. Higher pH may also have been associated with As desorption through competition between As oxyanions and OH- on mineral surfaces [34,35]. Therefore, the increase in organic carbon and decrease in Eh were associated with decreased CaCl_2_-Cd and increased KH_2_PO_4_-As, while amendment-specific changes in soil pH and Fe-associated fractions may have further contributed to the contrasting behavior of Cd/As in the amended paddy soil. Notably, FA generally produced greater increases in DOC and greater increases in KH_2_PO_4_-As, whereas HA caused a clear increase in soil pH and achieved greater reductions in CaCl_2_-Cd at higher application rates. These contrasting responses suggest that DOC-associated As mobilization may have been more pronounced under FA, while pH-mediated Cd immobilization may have contributed more strongly under HA [30,34,35]. Meanwhile, the concurrent changes in Eh and operationally defined Fe fractions indicate that Fe-associated processes may have interacted with both pathways rather than acting independently [20,33]. It should also be noted that the two amendments were commercial products with different physicochemical properties. FA was produced by ammonium sulfite digestion and FA-related responses may include not only humic-molecule effects but also potential N- and S-related inputs. Under flooded conditions, sulfur-mediated processes, including potential sulfide formation, may contribute to Cd immobilization [36], but sulfide and other sulfur species were not measured in this study. HA was applied as sodium humate, and HA-related responses may reflect both humic molecules and Na input or alkalinity associated with sodium humate. Because amendment N, S, and Na concentrations were not quantified and an alkalinity-matched control was not included, the specific role of the humic fraction could not be fully disentangled from these accompanying effects. Thus, the observed differences between FA and HA treatments should be interpreted as the integrated effects of the two commercial amendments and cannot be attributed solely to the intrinsic properties of FA and HA.

### 4.2. Effects of Humic Substance Amendments on Iron Plaque-Associated Cd/As Retention

The two humic substance amendments decreased Cd_IP_, whereas As_IP_ showed contrasting responses, decreasing under FA but increasing under HA (Figure 3). Thus, the effects of the two amendments differed more clearly for plaque-associated As than for plaque-associated Cd. Correlation analysis showed that Cd_IP_ was closely associated with soil CaCl_2_-Cd and the amounts of Fe and Mn in iron plaque (Figure 6). Previous studies have reported that decreases in soil available Cd may be associated with lower potential Cd supply to roots and reduced Cd retention on root surfaces [37,38]. Iron plaque is mainly composed of Fe/Mn (hydr)oxides and can provide abundant adsorption sites for metal(loid)s on rice root surfaces [19]. The changes in iron plaque formation and composition may influence Cd/As retention [39]. In this study, both Fe_IP_ and Mn_IP_ decreased with FA and HA and showed positive correlations with Cd_IP_ (Figure 3 and Figure 6), suggesting that changes in plaque-associated Fe/Mn pools were associated with the decline in Cd_IP_. However, iron plaque formation is also regulated by radial oxygen loss, root physiology, rice genotype, and Fe^2+^ supply [19], which were not directly characterized in this study. Therefore, the decrease in Fe_IP_ should not be attributed solely to amendment-induced changes in bulk-soil chemistry. Because Fe_IP_ and Mn_IP_ also varied among treatments, root-mass-normalized Cd_IP_ and As_IP_ should be interpreted as total plaque-associated Cd/As affected by both plaque abundance and metal(loid) enrichment, rather than as direct indicators of sorption or retention capacity alone. Thus, the decrease in Cd_IP_ indicates lower plaque-associated Cd, likely related to reduced plaque abundance and/or lower Cd exposure, rather than stronger plaque protection, as supported by the unchanged Cd_IP_/Fe_IP_ ratios (Figure S2).

In contrast, As_IP_ responded differently to the two humic amendments. In this study, As_IP_ with FA decreased and was negatively correlated with DOC, SOC, and KH_2_PO_4_-As (Figure 3c and Figure 6), indicating that the decrease in As_IP_ may be associated with FA-derived organic carbon and enhanced As mobilization. The increase in DOC may have been associated with stronger competition between dissolved organic ligands and As oxyanions for adsorption sites on the root surface [18], which could partly explain the lower plaque-associated As. Previous studies have shown that lower redox conditions may favor the reduction of As(V) to As(III), which is less strongly adsorbed than As(V) [40], but As speciation was not directly measured in this study. Although As_IP_ was not significantly correlated with Fe_IP_ under FA treatments (Figure 6), the decrease in Fe_IP_ may still have reduced the reactive plaque sites available for As retention. However, the As_IP_ increased with HA and was positively correlated with KH_2_PO_4_-As and soil pH (Figure 3d and Figure 6), suggesting that this response may be related to increased extractable As and pH-regulated As mobilization. This interpretation is consistent with previous studies showing that increased As mobility can increase the As pool available for retention by iron plaque, while higher pH may promote As desorption from soil minerals and thereby increase As mobility [22,41]. In addition, pH-dependent As adsorption on plaque-associated Fe phases may also have affected As retention, although this effect likely depends on As speciation, plaque surface properties, competing phosphate, and rhizosphere conditions [42], which were not directly characterized in this study. Therefore, the decrease in Cd_IP_ under FA and HA was associated with lower CaCl_2_-Cd and altered Fe/Mn plaque components, whereas the amendment-dependent responses of As_IP_ may be related to DOC-mediated competition, pH-associated As mobilization, and changes in KH_2_PO_4_-As.

### 4.3. Effects of Humic Substance Amendments on Rice Cd/As Concentrations, Distribution, and Health Risk

Both amendments decreased grain Cd concentration, with significant negative linear dose-response relationships under both FA and HA (Figure 4; Table S7). However, the magnitude of the reduction differed with application rate, indicating that neither amendment showed a consistent advantage across all doses. In contrast, grain As showed distinct dose-response patterns between the two amendments, decreasing linearly with increasing FA dose but showing a more complex rate-dependent response under HA. Grain Cd accumulation is generally controlled by soil availability, rhizosphere processes, root uptake, and internal translocation within rice plants [16,43]. In this study, correlation analysis showed that the grain Cd concentrations were related to CaCl_2_-Cd and Cd_IP_ (Figure 6), suggesting that extractable Cd and plaque-associated Cd were associated with grain Cd. Previous studies have also reported that decreasing soil available Cd and reducing Cd retention in iron plaque can lower Cd uptake and grain Cd accumulation in rice [44,45]. RF and linear regression analyses further identified that grain Cd was closely associated with DOC, Cd_IP_, SOC, and CaCl_2_-Cd (Figure 7 and Figure S3), suggesting that grain Cd was closely related to both soil Cd extractability and plaque-associated Cd. In addition, Cd translocation within rice plants is another important process controlling its final accumulation in grains [16]. In this study, Cd transfer factors showed amendment- and dose-dependent responses rather than consistent decreases (Figure S1), indicating changes in Cd concentration ratios among rice tissues. Similar reductions in grain Cd (76.62–97.01%) after organic or humic amendments have been reported in contaminated paddy soils [44], which is broadly comparable to the 30.95–87.68% decrease observed in the present study, although the underlying processes may vary with amendment type and soil conditions. Therefore, the decrease in grain Cd concentration under FA and HA was associated with decreased CaCl_2_-Cd and lower plaque-associated Cd, together with altered Cd distribution among rice tissues.

The As accumulation in rice is usually affected by soil As availability, As transformation, iron plaque retention, and translocation within plant tissues [45]. Soil available As is generally considered an important source for As uptake by rice, and higher As availability often promotes grain As accumulation [46]. In this study, however, the increase in KH_2_PO_4_-As with FA was not accompanied by enhanced As_IP_ or grain As concentrations (Figure 3 and Figure 4). These results reveal a decoupling between soil As extractability and grain As concentration, indicating that amendment-induced increases in KH_2_PO_4_-extractable As did not necessarily result in higher As concentration in rice grain. Previous studies have reported that root As retention may be related to cell wall binding, thiol complexation, and vacuolar sequestration [22,36], but these processes were not directly measured in this study. Thus, the decreased *TF*_root-stem_ and *TF*_stem-grain_ of As with FA (Figure S1) should be interpreted as changes in tissue As distribution. A different response was observed with HA. Grain As varied across HA application rates, with numerical increases at intermediate rates but no consistent increase at higher rates (Figure 4). Dose-response analysis detected no significant linear trend, while the significant quadratic term suggested curvature in the response; however, the overall quadratic model was only marginally non-significant (Table S7). Therefore, the HA response is more appropriately interpreted as a complex rate-dependent pattern rather than a clearly established non-monotonic dose-response pattern. Although extractable As was accompanied by higher As_IP_ under some HA treatments, grain As concentration was not strongly associated with either variable (Figure 6). Thus, the increase in grain As observed under HA2-HA3 cannot be directly attributed to As extractability or plaque-associated As alone. Previous studies have shown that As mobilization, plaque-associated retention, and internal redistribution may affect grain As accumulation [47,48]. In this study, the increased *TF*_root-stem_ and *TF*_stem-grain_ of As with HA indicated higher As concentration ratios between tissues (Figure S1), which was consistent with the increased grain As observed under some HA treatments. These patterns may be related to changes in As uptake pathways, xylem loading, phloem redistribution, or transporter activity [45,47], although these physiological mechanisms were not directly examined here. In addition, the *TF*-based changes did not translate into a monotonic increase in grain As across HA rates. Therefore, the contrasting grain As concentration responses to FA and HA appeared to reflect the combined influence of As mobilization, plaque-associated As, and tissue-level As distribution, although the relative contribution of these processes, especially under HA, remains uncertain. More importantly, the two amendments produced different Cd/As trade-offs across the tested application rates: both amendments reduced grain Cd concentration, whereas increasing FA dose was accompanied by a consistent decline in grain As, while HA produced a more variable grain As response across application rates.

The health risk assessment further highlighted the need to evaluate Cd and As simultaneously. The rice grains obtained from this pot experiment were experimental samples and were not intended for human consumption. Under the conservative screening-level exposure scenario, all *HI* values remained greater than 1. The decrease in grain Cd was accompanied by a clear reduction in *HQ*-Cd under both amendments, suggesting that FA and HA may reduce Cd-related dietary exposure (Figure 5). However, *HQ*-As was much higher than *HQ*-Cd and dominated HI across all treatments, indicating that reductions in *HQ*-Cd alone were insufficient to substantially lower the overall screening-level *HI*. Because inorganic As was not measured, these *HI* values should be interpreted as conservative screening-level indicators rather than absolute estimates of dietary health risk. In addition, cooking-related changes in grain Cd/As concentrations and gastrointestinal bioaccessibility were not considered, which may further affect actual dietary exposure. Similar findings have been reported in Cd/As co-contaminated rice systems, where As contributed more strongly than Cd to non-carcinogenic risk estimates through rice consumption [3,17]. Therefore, although FA6 and HA6 produced the largest reductions in grain Cd concentration, they should not be regarded as optimum amendment rates based on contaminant concentrations alone, because grain As responses, overall screening-level health risk, plant biomass, grain yield, economic feasibility, possible N and S inputs from the FA, and potential Na inputs from sodium humate also need to be considered.

Several limitations should be acknowledged. This study was conducted using a single Cd/As-contaminated soil and one rice cultivar under controlled pot conditions. In addition, biomass and grain yield were not measured; therefore, the observed changes in tissue and grain Cd/As concentrations cannot be directly translated into changes in total plant accumulation or agronomic productivity. Accordingly, the present study focuses on Cd/As concentrations and concentration-based distribution across the soil–rice system, and field validation incorporating plant growth and yield responses is needed before practical amendment recommendations can be made.

## 5. Conclusions

A pot experiment was conducted with two commercial humic-substance amendments, fulvic acid (FA) and humic acid (HA), to evaluate their effects on Cd/As behavior in a Cd/As co-contaminated paddy soil–rice system. Both FA and HA decreased CaCl_2_-Cd and grain Cd concentration while increasing KH_2_PO_4_-As. This reduction in CaCl_2_-Cd was accompanied by Cd redistribution toward less labile fractions, whereas the increase in KH_2_PO_4_-As was associated with increased soluble and Al-bound As and decreased Fe-bound As. Grain As concentration showed amendment- and dose-dependent responses, with decreases at higher FA rates but increases at some HA rates. These grain As responses were associated with differences in plaque-associated As and concentration-based transfer factors. Correlation and Random Forest analyses suggested that grain Cd/As variation was associated with DOC, plaque-associated metals, pH, and extractable metal(loid) pools. The conservative screening-level health risk assessment indicated that *HQ*-As was the main contributor to *HI*, and all *HI* values remained above 1 despite reductions in the *HQ*-Cd; however, these values may overestimate As-related risk because total As rather than inorganic As was used in the calculation and should not be interpreted as evidence of actual dietary health risk. Overall, the two humic amendments produced distinct and dose-dependent Cd/As trade-offs across the soil–rice system. Both amendments reduced grain Cd concentration, but their combined Cd/As responses differed across the tested application rates. Higher FA rates reduced both grain Cd and grain As, whereas intermediate HA rates increased grain As despite concurrent reductions in grain Cd. Importantly, the increase in KH_2_PO_4_-As did not consistently translate into increased grain As, demonstrating that soil extractability alone is insufficient to predict the As concentration in rice grain. These findings provide evidence that the concentration-based responses of Cd and As in amended paddy soils depend jointly on amendment type, application rate, root-surface association, and tissue-level distribution.

## Figures and Tables

**Figure 1:**
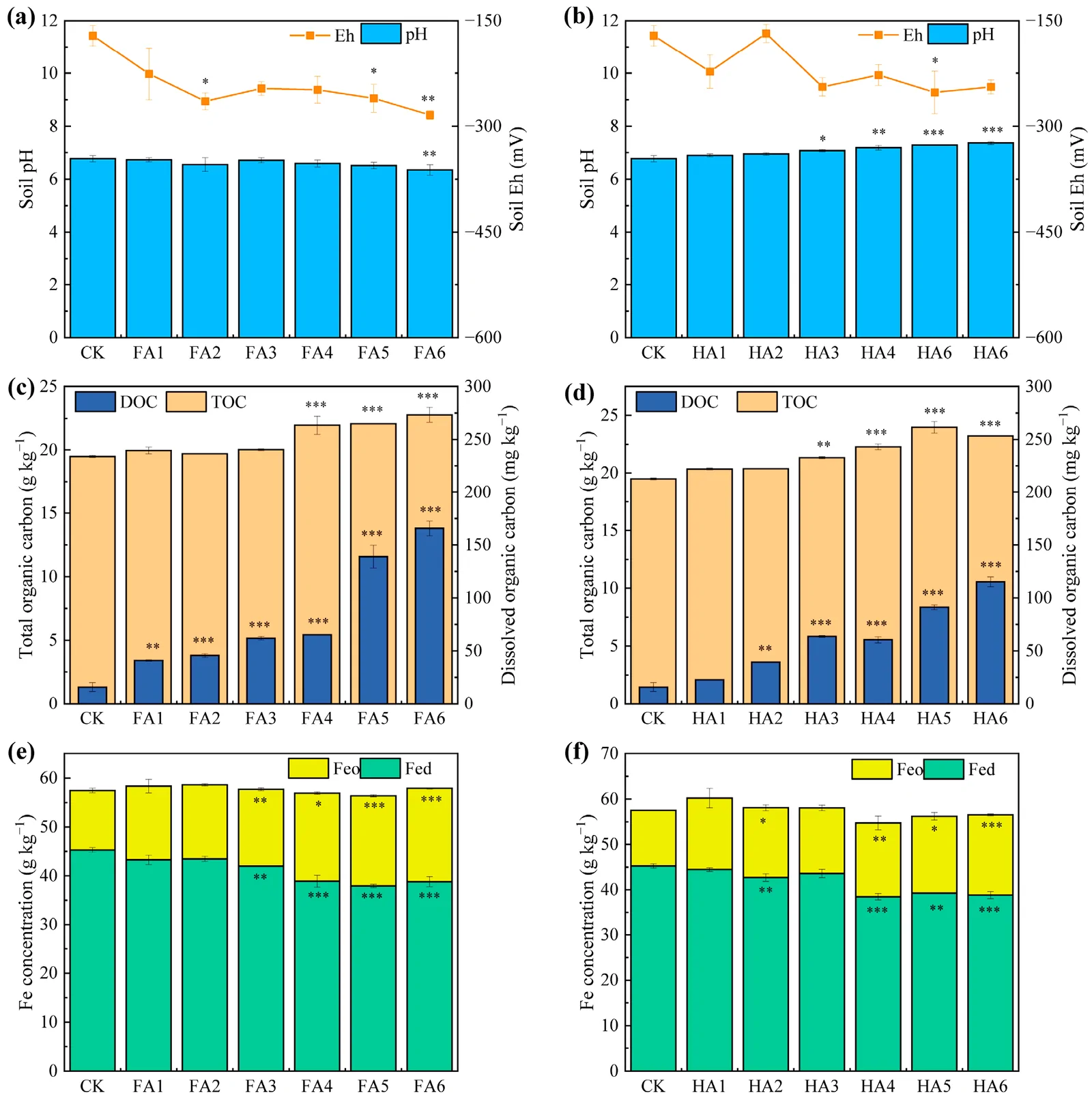
Changes in soil pH and Eh (**a**,**b**), total and dissolved organic carbon (**c**,**d**), and Fe oxide fractions (**e**,**f**) under FA and HA amendments. Fed and Feo denote dithionite–citrate–bicarbonate-extractable Fe and acid ammonium oxalate-extractable Fe, respectively. Data are means ± SE (*n* = 3). Error bars represent SEs. Asterisks indicate significant differences from the shared CK based on Dunnett-adjusted *p* values: * *p* < 0.05, ** *p* < 0.01, and *** *p* < 0.001.

**Figure 2:**
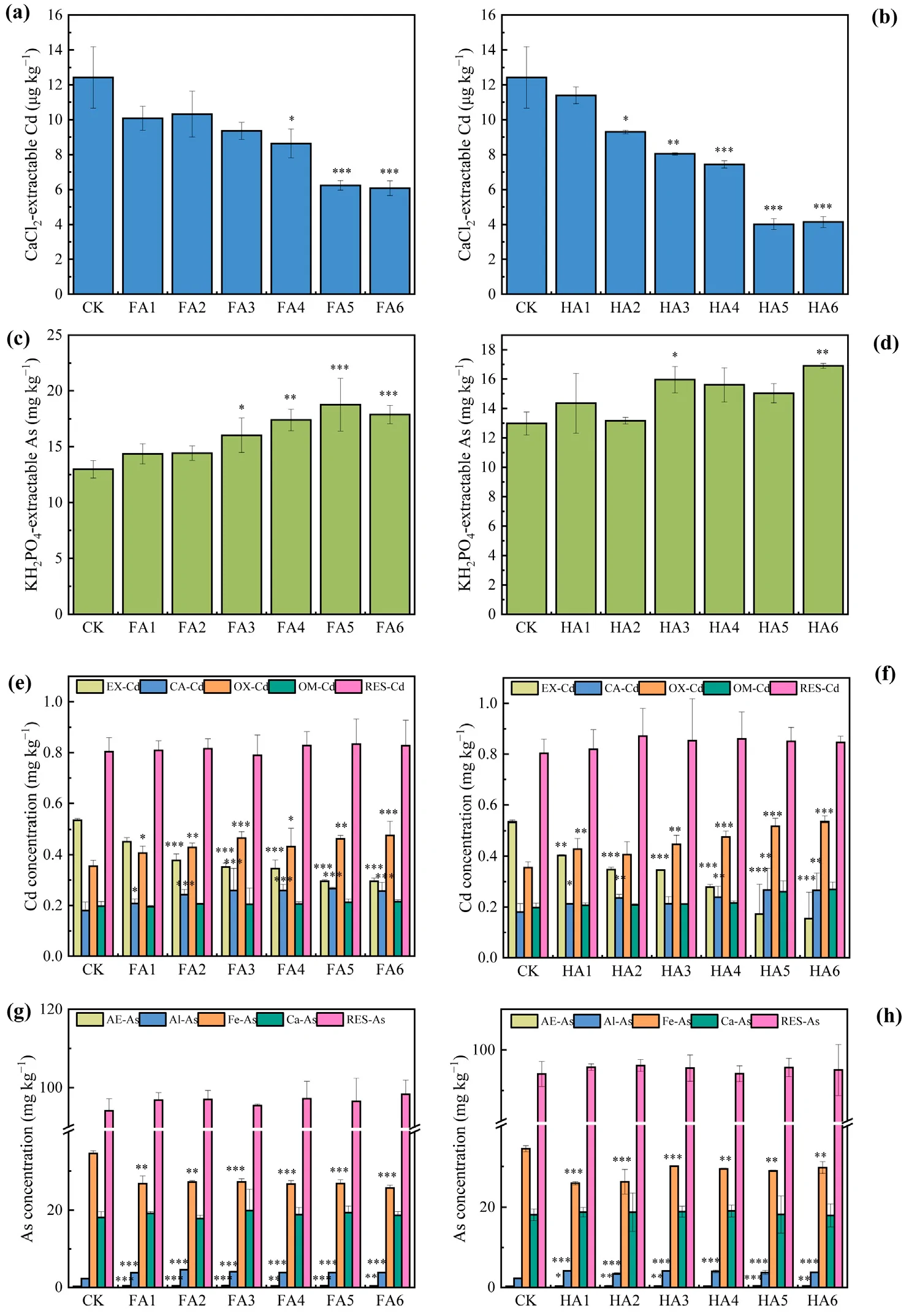
Changes in soil extractable Cd/As and Cd/As fractions under humic substance amendments. CaCl_2_-extractable Cd, KH_2_PO_4_-extractable As, Cd fractions, and As fractions are shown in (**a**,**b**), (**c**,**d**), (**e**,**f**), and (**g**,**h**), respectively. Data are means ± SE (*n* = 3). Error bars indicate SEs. Asterisks indicate significant differences from the shared CK based on Dunnett-adjusted *p* values: * *p* < 0.05, ** *p* < 0.01, and *** *p* < 0.001.

**Figure 3:**
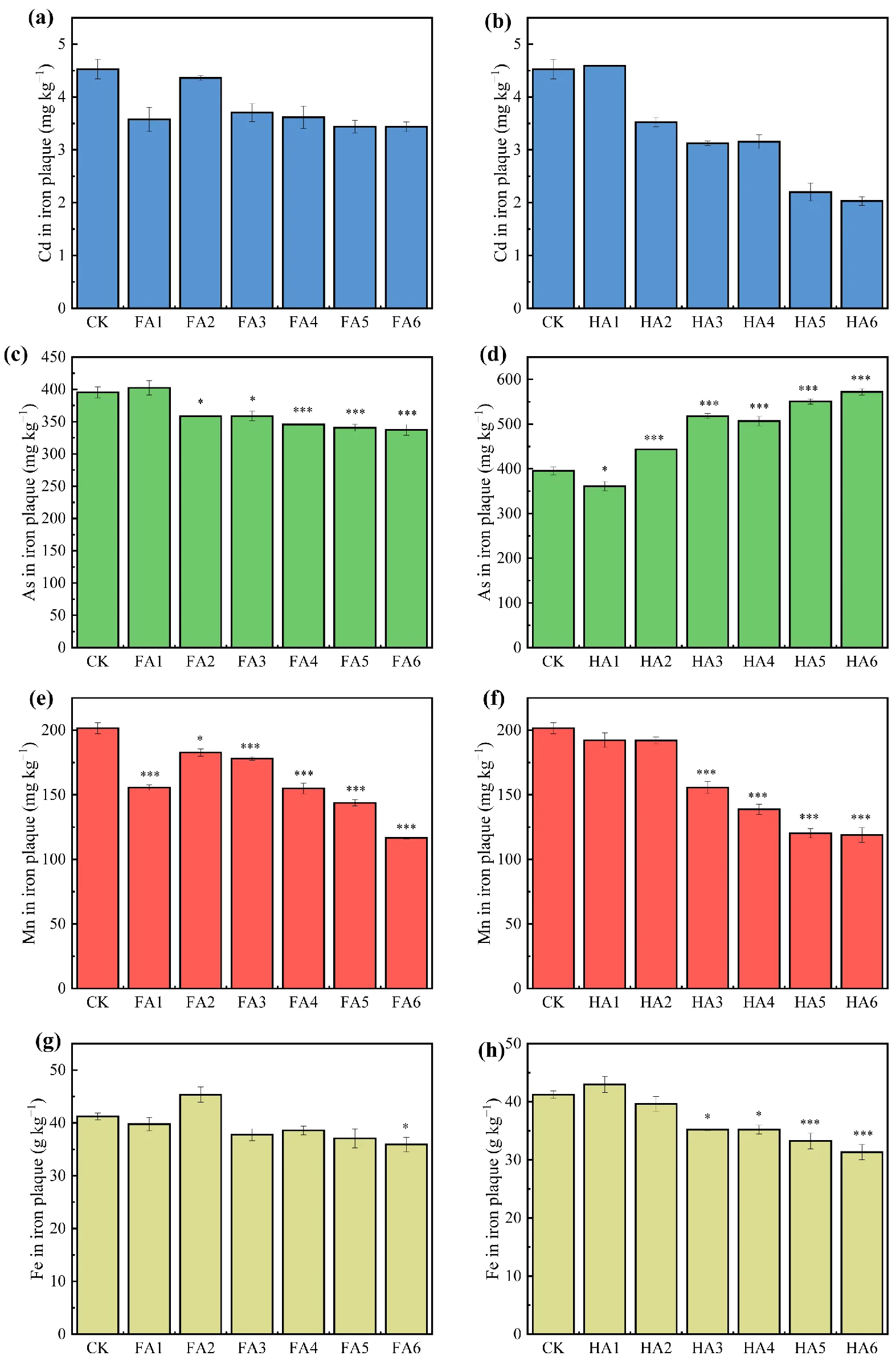
Changes in Cd (**a**,**b**), As (**c**,**d**), Mn (**e**,**f**), and Fe (**g**,**h**) in iron plaque under humic substance amendments. Data are means ± SE (*n* = 3). Error bars indicate SEs. Asterisks indicate significant differences from the shared CK based on Dunnett-adjusted *p* values: * *p* < 0.05, ** *p* < 0.01, and *** *p* < 0.001.

**Figure 4:**
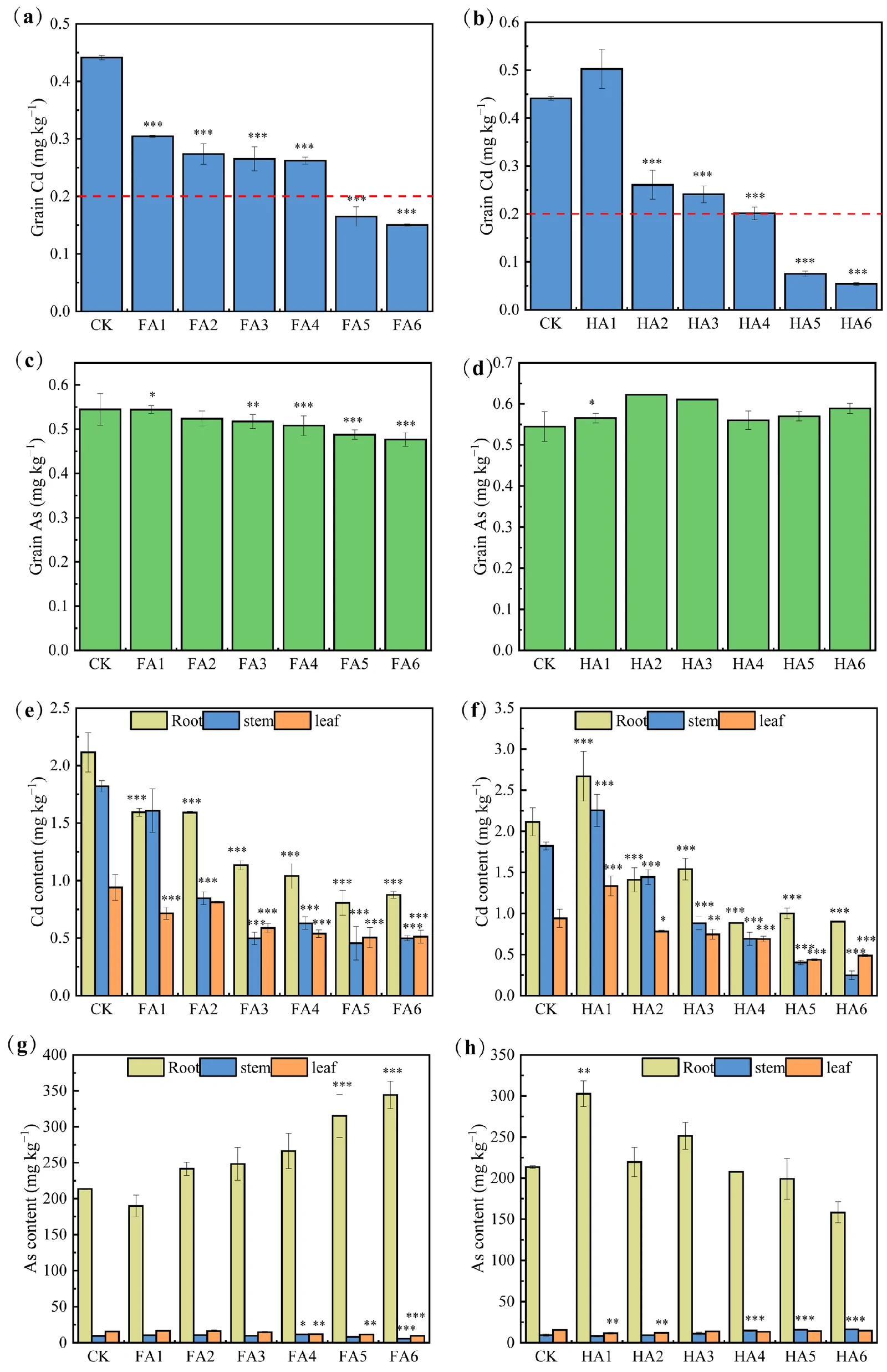
Effects of humic substance amendments on Cd and As concentrations in rice tissues. Grain Cd concentrations under FA and HA treatments are shown in (**a**,**b**), respectively, and grain As concentrations are shown in (**c**,**d**), respectively. Cd concentrations in vegetative tissues are shown in (**e**,**f**), and As concentrations in vegetative tissues are shown in (**g**,**h**). The red dashed line in the grain Cd panels represents the Cd limit of 0.2 mg kg^−1^ specified for rice in [27] and is provided as an approximate reference. No regulatory reference line was added for grain As because total As rather than inorganic As was determined in this study. Data are means ± SE (*n* = 3). Error bars represent SEs. Asterisks indicate significant differences from the shared CK based on Dunnett-adjusted *p* values: * *p* < 0.05, ** *p* < 0.01, and *** *p* < 0.001.

**Figure 5:**
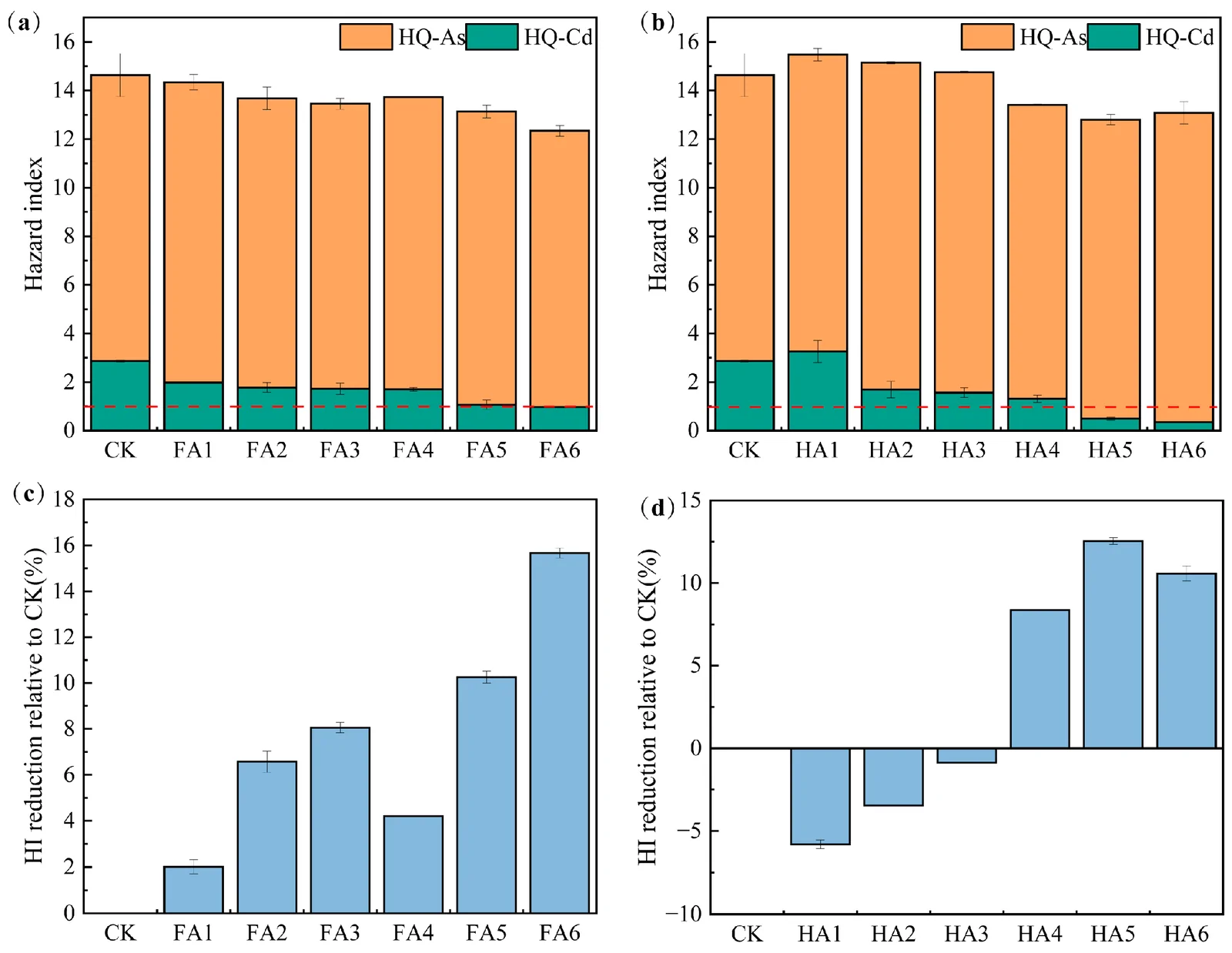
Effects of humic substance amendments on potential health risks of Cd and As exposure through rice consumption. *HQ*-Cd and *HQ*-As under FA and HA treatments are shown in (**a**,**b**), respectively, with the total stacked height representing *HI*. Changes in *HI* relative to CK under FA and HA treatments are shown in (**c**,**d**), respectively. *HQ*-As and HI were calculated under the conservative assumption that total grain As was equivalent to inorganic As. The dashed red lines in (**a**,**b**) indicate the *HI* reference value of 1. Data are means ± SE (*n* = 3). Error bars represent SEs.

**Figure 6:**
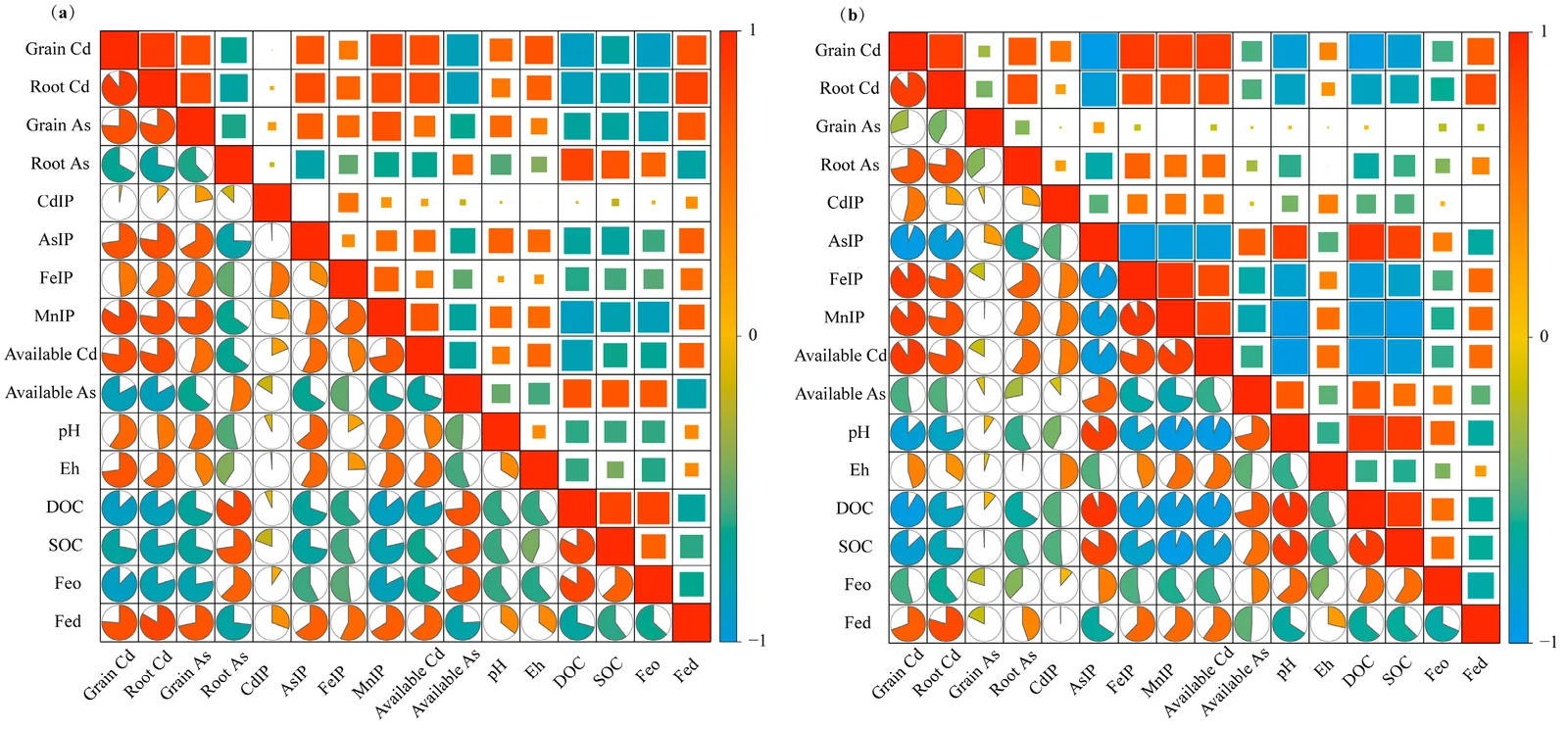
Amendment-specific correlations among soil properties, extractable Cd/As, metals in iron plaque, and Cd/As in rice tissues. Correlations under FA and HA treatments are shown in (**a**,**b**), respectively. Pearson’s correlation coefficients were calculated using individual pot-level observations. The shared CK observations were included in both amendment-specific datasets (*n* = 21 for each analysis). Statistical significance was evaluated after Benjamini–Hochberg false discovery rate (FDR) correction for multiple comparisons.

**Figure 7:**
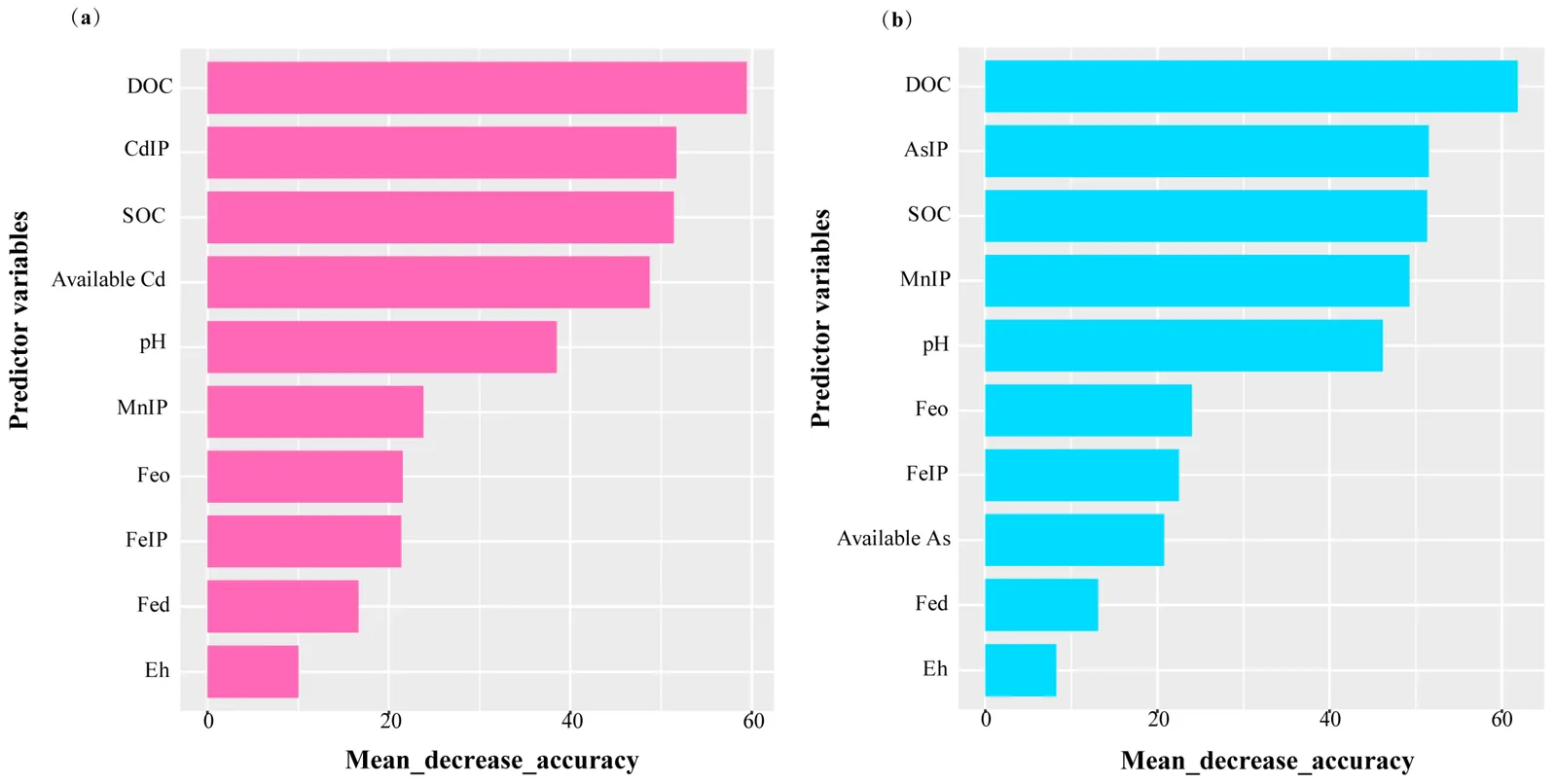
Random Forest analysis ranking variables associated with grain Cd and As concentrations under humic substance amendments. Variable importance for grain Cd and grain As is shown in (**a**,**b**), respectively. Variable importance was evaluated using the mean decrease in accuracy based on individual pot-level observations (*n* = 39).

**Table 1:** Carbon-equivalent doses and corresponding actual application rates of FA and HA amendments.

Treatment	Carbon-Equivalent Dose (g C kg^−1^ Soil)	FA Application Rate (g kg^−1^ Soil)	HA Application Rate (g kg^−1^ Soil)	FA Mass per Pot (g Pot^−1^)	HA Mass per Pot (g Pot^−1^)
CK	0	0	0	0	0
FA1/HA1	0.5	1.77	1.64	8.85	8.19
FA2/HA2	1	3.54	3.27	17.71	16.37
FA3/HA3	2	7.08	6.55	35.41	32.74
FA4/HA4	3	10.62	9.82	53.12	49.12
FA5/HA5	4	14.16	13.10	70.82	65.49
FA6/HA6	5	17.71	16.37	88.53	81.86

Note: The actual amendment masses were calculated from the carbon-equivalent doses according to the TOC concentrations of FA (28.24%) and HA (30.54%). Each pot contained 5 kg of soil. For approximate field-scale comparison, assuming 150,000 kg soil per mu for the 0–20 cm layer, the FA6 and HA6 rates correspond to approximately 39.8 and 36.8 t ha^−1^, respectively.

## Data Availability

The raw dataset used for the statistical analyses is provided as Data S1 in the Supplementary Materials.

## Supplementary Materials

The following supporting information can be downloaded at https://www.mdpi.com/article/10.3390/toxics14100842/s1: Method SI: Determination methods of soil pH, Eh, total Cd/As, and available Cd/As; Method SII: Determination methods of iron/manganese oxide fractions; Method SIII: Detailed procedures of the Cd and As sequential extractions; Method SIV: Determination methods of iron plaque and Cd/As in rice tissues; Method SV: Determination methods of SOC and DOC. Figure S1: Changes in concentration-based translocation factors (*TF*) of Cd and As in rice under humic substance amendments. *TF*_stem-grain_ of Cd, *TF*_stem-grain_ of As, *TF*_root-stem_ of Cd, and *TF*_root-stem_ of As are shown in (a,b), (c,d), (e,f), and (g,h), respectively. Data are means ± SE (*n* = 3). Error bars represent SEs. Different letters indicate significant differences among treatments at *p* < 0.05; Figure S2: Fe-normalized plaque-associated Cd and As under FA and HA treatments. Cd_IP_/Fe_IP_ (a,b) and As_IP_/Fe_IP_ (c,d) were calculated from replicate-level DCB-extractable Cd, As, and Fe values normalized to root dry weight. Data are means ± SE (*n* = 3). Error bars indicate SEs. Different lowercase letters indicate significant differences among treatments at *p* < 0.05; Figure S3: Regression relationships between selected predictors and grain Cd/As (*n* = 3). Relationships between grain Cd and dissolved organic carbon (DOC), Cd in iron plaque (Cd_IP_), and CaCl_2_-extractable Cd are shown in (a), (c), and (e), respectively. Relationships between grain As and dissolved organic carbon (DOC), As in iron plaque (As_IP_), and KH_2_PO_4_-extractable As are shown in (b), (d), and (f), respectively. Table S1: Estimated daily intake, hazard quotient, and hazard index of Cd and As through rice consumption under different humic substance amendments. Data are means ± SE (*n* = 3); Table S2: QA/QC information and instrumental performance parameters for Cd, As, Fe, and Mn determination; Table S3: Mean ± SE values of soil properties, extractable Cd, Cd fractions, plaque-associated metals, rice tissue Cd contents under different treatments. Data are means ± SE (*n* = 3); Table S4: Mean ± SE values of soil properties, extractable As, As fractions, plaque-associated metals, rice tissue As contents under different treatments. Data are means ± SE (*n* = 3); Table S5: Effects of amendment type, carbon-equivalent dose, and their interaction on soil and rice variables; Table S6: Dose-specific comparisons between FA and HA treatments under carbon-equivalent application rates; Table S7: Amendment-specific dose-response relationships for selected soil and grain variables under carbon-equivalent application rates. Data S1: Raw pot-level dataset used for statistical analyses.
