# Supplementary material for: Effects of Humic Substance Amendments on Cadmium and Arsenic Availability, Concentrations, and Potential Health Risk in Rice
Source: Toxics. 2026 Sep 22;14(10):842. doi: 10.3390/toxics14100842 (PMC13709761; doi:10.3390/toxics14100842)
Supplement: Supplementary file 1 [file toxics-14-00842-s001.zip › toxics-4539604-supplementary.pdf]

Effects of Humic Substance Amendments on Cadmium and Arsenic Availability, Concentrations, and Potential Health Risk in Rice

Method SI. Determination methods of soil pH, Eh, total Cd / As, and available Cd/As

The soil redox potential (Eh) was initially measured at 10 cm below the soil surface using a redox potential depolarization automatic analyzer (FJA, Chuan-Di Instrument & Equipment Co., Ltd., Nanjing, China). Soil pH was measured in a soil-water ratio of 1:2.5 (wt/vol) using a pH meter (PHS-3C, Shanghai INESA Scientific Instrument Co., Ltd., Shanghai, China). Total Cd and As concentrations in soil and organic amendments were digested by HCl-HNO_3 (4:1, v/v) with an open system. The soil available Cd and As were extracted by the 0.01 mol L^{-1} $CaCl_2$ (1:5, w/v) and 0.5 mol L^{-1} KH_2PO_4 (1:15 w / v), respectively, with 120 min shaking. The Cd and As concentrations in all the above digested and extracted supernatants were determined by ICP-OES (5110, Agilent Technologies, Santa Clara, CA, USA) and atomic fluorescence spectrophotometry (AFS-8530, Beijing Haiguang Instrument Co., Ltd., Beijing, China). Quality control using reference soil GBW-070011 (National Standard Soil) yielded Cd and As recoveries of 87.2%–113.5% and 90.1%–107.7%.

Method SII. Determination methods of iron/manganese oxide fractions

Dithionite–citrate–bicarbonate-extractable Fe (Fed) were extracted using the DCB method, whereas acid ammonium oxalate-extractable Fe (Feo) were extracted with 0.2 mol L^{-1} ammonium oxalate. These fractions were operationally defined extractable Fe and Mn pools rather than discrete crystalline or amorphous oxide phases. Fe concentrations in the extracts were determined by ICP-OES (5110, Agilent Technologies, Santa Clara, CA, USA). External calibration was performed using certified Fe standard solutions, and procedural blanks and duplicate samples were included for quality control.

Method SIII. Detailed procedures of the Cd and As sequential extractions

The speciation of Cd was determined by the modified sequential extraction method according to Sut-Lohmann et al. (2022). The five extraction procedures were as follows.

Exchangeable Cd plus water-soluble Cd (EX-Cd): 1 g soil and 25 mL $MgCl_2$ (1mol L^{-1}) were mixed into each 100 mL centrifuge tube and shaken at 180 rpm for 2 h at 25°C. The tubes were then centrifuged for 10 min at 4000 rpm, and the supernatants were collected.

Carbonate-bound Cd (CA-Cd): 25 mL $NaAC_2$ (1 mol L^{-1}, pH = 5.0) was mixed into the aforementioned tubes and then shaken at180 rpm for 5 h at 25°C.

Iron (manganese) oxide bound Cd (OX-Cd): 50 mL NH_2OH-HCl and HCl mixture (0.25 mol L^{-1}) was mixed into the aforementioned tubes and then shaken at 180 rpm for 6h at 25°C.

Organic bound Cd (OM-Cd): 5 mL H_2O_2 (30%) and 3 mL HNO_3 were mixed into the aforementioned tubes and maintained in a water bath at 83°C for 90 min, and the above step was repeated after 3 mL H_2O_2 addition, followed by diluted to 25 ml.

Residual Cd (RES-Cd): The residual soil in the tubes was dried and digested using mixed acids of HCl-HNO_3-$HClO_4$. Three samples were prepared for each treatment and analyzed by ICP-OES (5110, Agilent Technologies, Santa Clara, CA, USA).

The speciation of As was determined by the modified sequential extraction method according to Luo et al. (2024). The five extraction procedures were as follows.

Soluble As (AE-As): 1 g soil and 50 mL NH_4Cl (1 mol L^{-1}) was mixed into each 100 mL centrifuge tube and shaken at 180 rpm for 0.5 h at 25°C. The tubes were then centrifuged for 10 min at 4000 rpm, and the supernatants were collected.

Al-bound As (Al-As): 50 mL NH_4F (0.5 mol L^{-1}) was mixed into the aforementioned tubes and then shaken at 180 rpm for 1 h at 25°C.

Fe-bound As (Fe-As): 50 mL NaOH (0.1 mol L^{-1}) was mixed into the aforementioned tubes, shaken at 180 rpm for 2 h at 25°C and then shaken for 2 h after waiting 16 h.

Ca-bound As (Ca-As): 50 mL H_2SO_4 (0.25 mol L^{-1}) was mixed into the aforementioned tubes and shaken at 180 rpm for 1 h at 25°C.

Residual As (RES-As): The residual soil in the tubes was dried and digested using mixed acids of HCl-HNO_3-$HClO_4$. Three samples were prepared for each treatment and analyzed by ICP-MS (7900, Agilent Technologies, Santa Clara, CA, USA).

The recoveries of the sequential extraction procedure, calculated as the sum of the operationally defined fractions divided by the independently measured total Cd or As concentration, were 92.3%–105.2% for Cd and 89.7%–112.4% for As, respectively.

Method SIV. Determination methods of iron plaque and Cd/As in rice tissues

Iron plaque on rice roots was extracted using the dithionite-citrate-bicarbonate (DCB) method. Briefly, fresh root subsamples were gently cleaned to remove adhering soil particles and rinsed with deionized water. Approximately 2.0 g of fresh roots was immersed in 40.0 mL of DCB solution and extracted for 10 min. Then, 1.0 g of sodium dithionite was added, and the roots were extracted for another 50 min at room temperature. The extract was transferred to a 100 mL volumetric flask and diluted to volume with deionized water. After filtration, Fe and Mn concentrations in the DCB extracts

were determined by ICP-OES (5110, Agilent Technologies, Santa Clara, CA, USA), while Cd and As concentrations were measured using an ICP-MS (7900, Agilent Technologies, Santa Clara, CA, USA). Plaque-associated Fe, Mn, Cd, and As were normalized to root dry weight. After DCB extraction, the roots were rinsed with deionized water, dried, ground, and then used for root tissue Cd/As analysis..

Cd and As in rice tissues were determined after microwave-assisted digestion using HNO_3-H_2O_2 (8:1, v/v), followed by dilution to 10 mL with 1% HNO_3 in colorimetric tubes prior to analysis. Quality control was performed using the certified reference material bush twigs and leaves (GBW-07602), with Cd and As recoveries ranging from 96.4% to 105.3% and from 92.1% to 99.5%, respectively.

Method SV. Determination methods of SOC and DOC

Total organic carbon (TOC) was determined using $K_2Cr_2O_7$ oxidation-$FeSO_4$ titration method. Dissolved organic carbon (DOC) was extracted with ultrapure water at a soil-to-water ratio of 1:5 (w/v) and quantified using a TOC analyzer (TOC-VWP, Shimadzu Corporation, Kyoto, Japan).

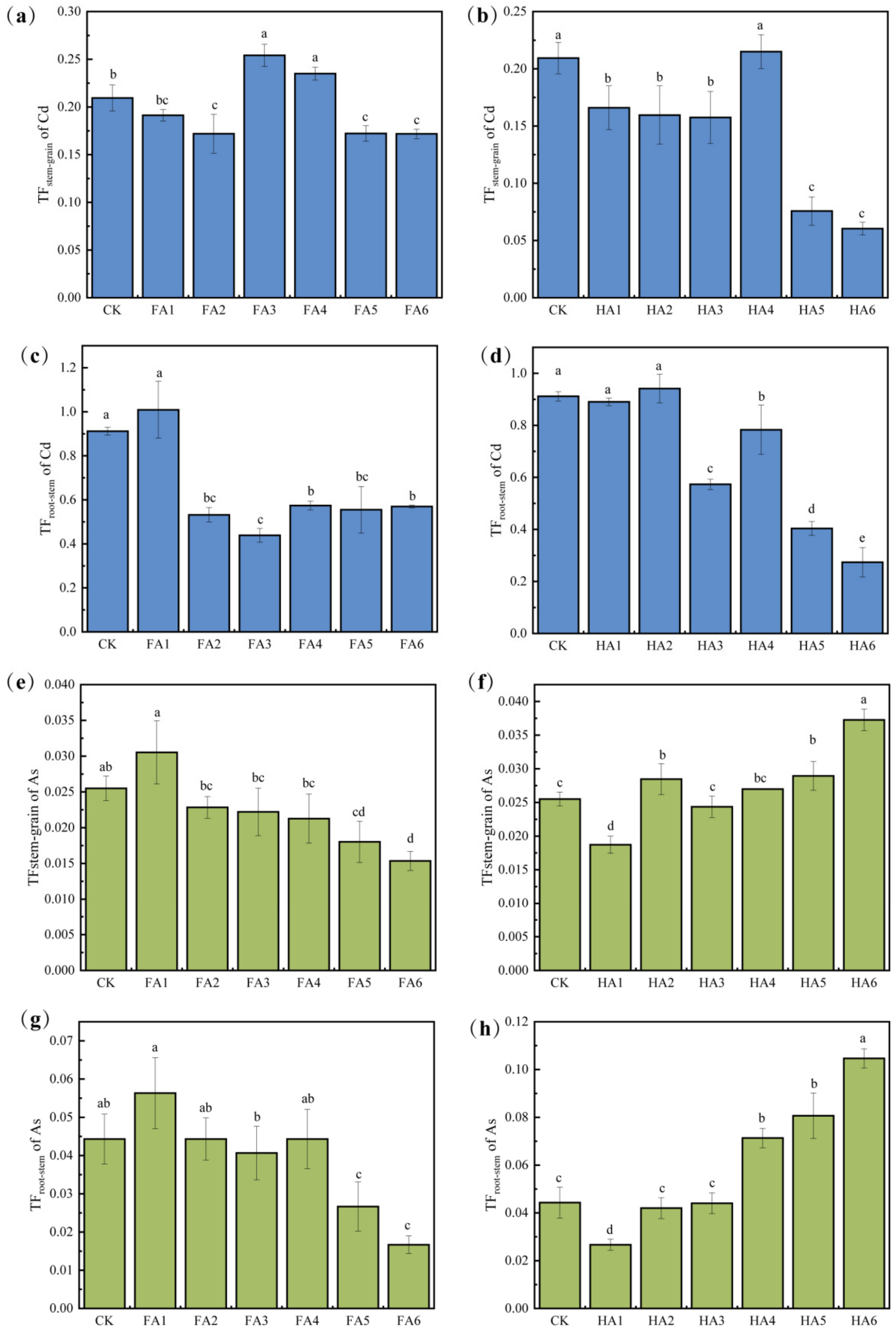

Figure S1. Changes in concentration-based translocation factors (*TF*) of Cd and As in rice under humic substance amendments. $TF_{\text{stem-grain}}$ of Cd, $TF_{\text{stem-grain}}$ of As, $TF_{\text{root-stem}}$ of Cd, and $TF_{\text{root-stem}}$ of As are shown in (a, b), (c, d), (e, f), and (g, h), respectively. Data are means ± SE (n = 3). Error bars represent SEs. Different letters indicate significant differences among treatments at P<0.05.

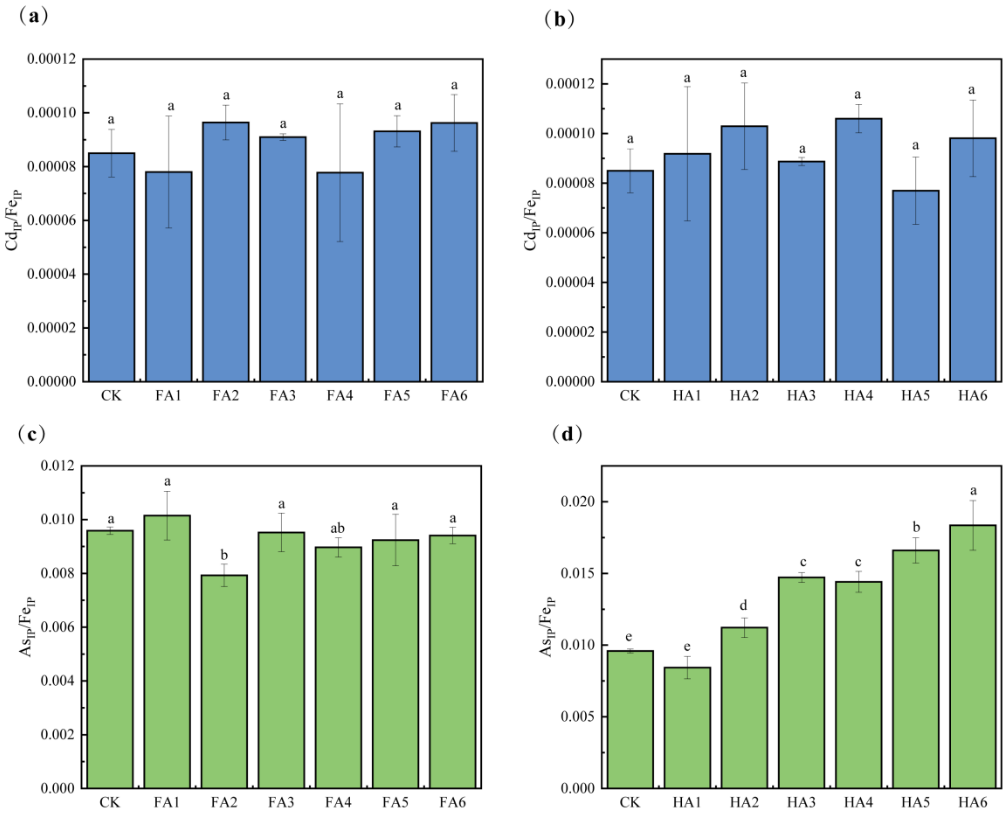

Figure S2. Fe-normalized plaque-associated Cd and As under FA and HA treatments. Cd_{IP}/Fe_{IP} (a, b) and As_{IP}/Fe_{IP} (c, d) were calculated from replicate-level DCB-extractable Cd, As, and Fe values normalized to root dry weight. Data are means ± SE (n = 3). Error bars indicate SEs. Different lowercase letters indicate significant differences among treatments at $P < 0.05$.

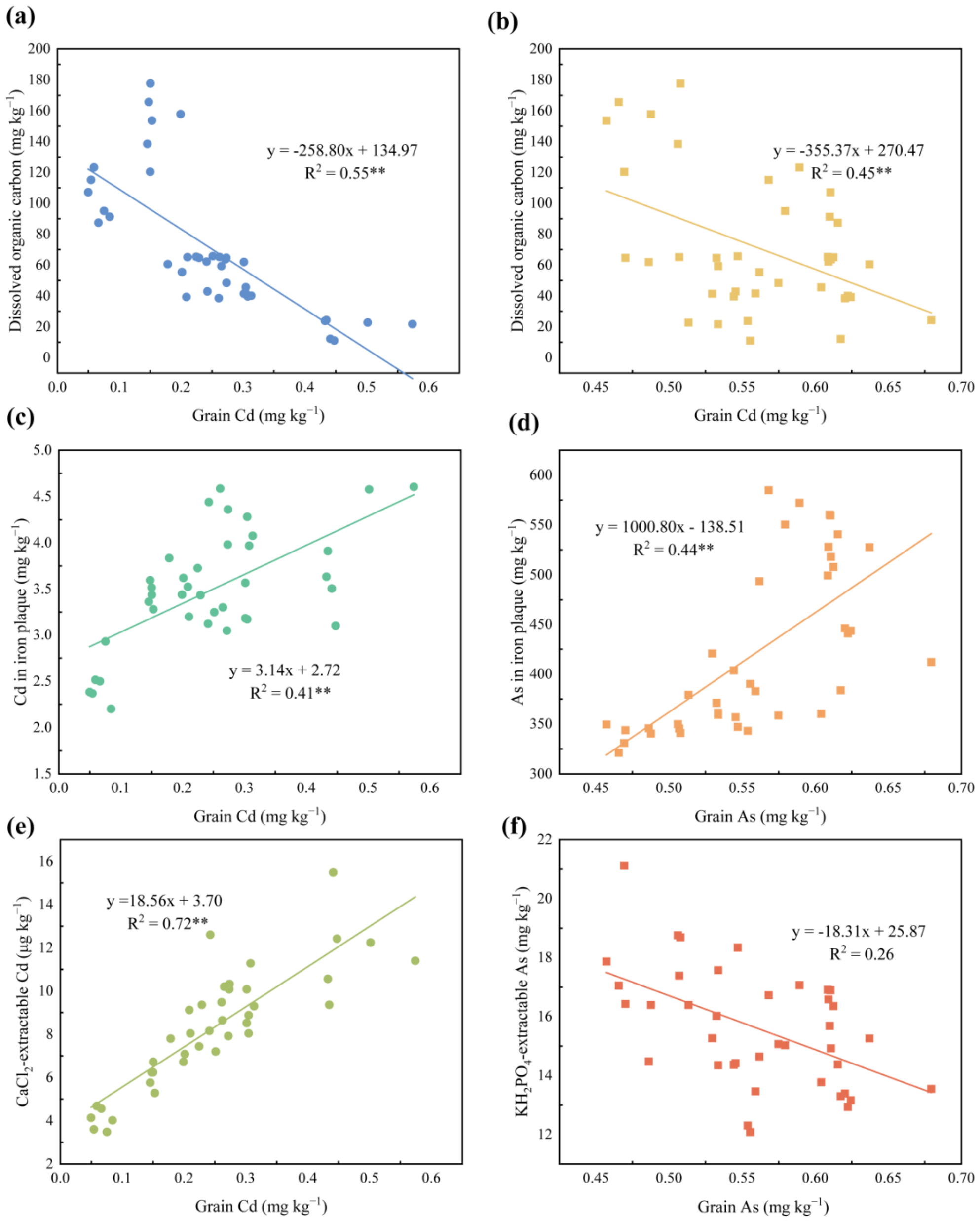

Figure S3. Regression relationships between selected predictors and grain Cd/As (n = 3). Relationships between grain Cd and dissolved organic carbon (DOC), Cd in iron plaque (Cd_{IP}), and $CaCl_2$-extractable Cd are shown in (a), (c), and (e), respectively. Relationships between grain As and dissolved organic carbon (DOC), As in iron plaque (As_{IP}), and KH_2PO_4-extractable As are shown in (b), (d), and (f), respectively.

Table S1. Estimated daily intake, hazard quotient, and hazard index of Cd and As through rice consumption under different humic substance amendments. Data are means ± SE (n = 3).

Treatment	Grain Cd<br>mg kg^{-1}	Grain As<br>mg kg^{-1}	*EDI*-Cd<br>mg kg-1 BW day^{-1}	*EDI*-As<br>mg kg-1 BW day^{-1}	*HQ*-Cd	*HQ*-As	*HI*
CK	0.44±0.01	0.54±0.04	0.003±0.00006	0.004±0.0003	2.86±0.04	11.77±0.88	14.63±0.87
FA1	0.30±0.01	0.57±0.01	0.002±0.00001	0.004±0.0001	1.98±0.02	12.36±0.31	14.34±0.33
FA2	0.27±0.03	0.55±0.02	0.002±0.0002	0.004±0.0001	1.77±0.20	11.90±0.46	13.67±0.62
FA3	0.27±0.04	0.54±0.01	0.002±0.0003	0.004±0.0001	1.72±0.24	11.74±0.23	13.46±0.40
FA4	0.26±0.02	0.56±0.06	0.002±0.0001	0.004±0.0001	1.70±0.07	12.04±0.01	13.74±0.07
FA5	0.16±0.01	0.53±0.01	0.001±0.0002	0.004±0.0001	1.07±0.19	12.06±0.26	13.13±0.45
FA6	0.15±0.01	0.57±0.01	0.001±0.00001	0.003±0.0001	0.97±0.02	11.37±0.22	12.34±0.23
HA1	0.50±0.07	0.50±0.07	0.003±0.0005	0.004±0.0001	3.26±0.46	12.22±0.26	15.48±0.71
HA2	0.26±0.05	0.62±0.02	0.002±0.0003	0.004±0.0001	1.69±0.34	13.45±0.04	15.14±0.32
HA3	0.24±0.03	0.61±0.02	0.002±0.0002	0.004±0.0001	1.56±0.20	13.20±0.04	14.76±0.18
HA4	0.20±0.02	0.56±0.03	0.001±0.0002	0.004±0.0001	1.31±0.15	12.11±0.03	13.41±0.14
HA5	0.08±0.01	0.57±0.01	0.0005±0.00006	0.004±0.0001	0.49±0.06	12.31±0.21	12.80±0.19
HA6	0.05±0.01	0.59±0.02	0.0004±0.00006	0.004±0.0001	0.35±0.03	12.73±0.46	13.09±0.44

Table S2. QA/QC information and instrumental performance parameters for Cd, As, Fe, and Mn determination.

Analytical item	Instrument	QA/QC or instrumental performance
Soil Cd and As analysis	ICP-MS (Agilent 7900, USA)	Procedural blanks were included during soil digestion and extraction. Procedural blanks were included during soil digestion and extraction. All procedural blank concentrations were below the corresponding method detection limits. Reference soil GBW-070011 was used for accuracy control, with recoveries of 87.2%–113.5% for Cd and 90.1%–107.7% for As.
Sequentially extracted Cd and As fractions	ICP-MS (Agilent 7900, USA)/ ICP-OES (Agilent 5110, USA)	The recoveries of the sequential extraction procedures were 92.3%–105.2% for Cd and 89.7%–112.4% for As. These recoveries were calculated from the sum of extracted fractions relative to total Cd or As.
Rice tissue Cd and As analysis	ICP-MS (Agilent 7900, USA)	Certified reference material GBW-07602 was used for plant sample quality control, with recoveries of 96.4%–105.3% for Cd and 92.1%–99.5% for As.
Cd and As determination	ICP-MS (Agilent 7900)	Cd/As were determined using the ICP-MS. The instrument specifications report 3σ detection limits at the ppt level and short- and long-term stability of RSD ≤ 2% and ≤ 3%, respectively. The method detection limits used for Cd and As in this study were 0.001 mg kg^{-1}.
Fe and Mn determination	ICP-OES (Agilent 5110, USA)	Fe in Fed/Feo extracts and Fe/Mn in DCB extracts of root iron plaque were determined using the ICP-OES. The instrument specifications report long-term stability of RSD ≤ 1% and replicate-measurement precision of RSD ≤ 0.5%. The method detection limits used for Fe and Mn in this study were 0.01 mg kg^{-1}.
Calibration and replicate measurements	ICP-MS (Agilent 7900, USA)/ICP-OES (Agilent 5110, USA)	External calibration was performed using certified standard solutions, with calibration coefficients greater than 0.999. Calibration verification standards were included during instrumental analysis. Each sample solution was measured in triplicate, and the mean of the three readings was used as the reported value. No sample concentrations were below the corresponding method detection limits; therefore, no substitution or censoring procedure was required.

Note: Method quantification limits (LOQs) were not independently established in this study; therefore, only the experimentally determined method detection limits (MDLs) are reported.

Table S3. Mean ± SE values of soil properties, extractable Cd, Cd fractions, plaque-associated metals, rice tissue Cd contents under different treatments. Data are means ± SE (n = 3).

	**StemCd**	**LeafCd**	**RootCd**	**CdIP**	**FeIP**	**MnIP**	**AvailableCd**	**EXCd**	**CACd**	**OXCd**	**pH**	**Eh**	**DOC**	**SOC**	**Feo**	**Fed**
CK	1.82±0.05	0.94±0.11	2.11±0.17	3.51±0.41	41.23±1.10	201.56±7.48	12.42±3.06	0.22±0.00	0.08±0.01	0.19±0.01	6.77±0.12	-171.15±25.45	15.85±7.39	19.49±0.15	12.21±0.08	49.79±0.54
FA1	1.61±0.19	0.72±0.05	1.59±0.03	3.11±0.90	39.77±2.18	155.61±3.67	10.08±1.20	0.20±0.01	0.10±0.00	0.23±0.01	6.73±0.07	-225.80±63.92	40.92±1.09	19.96±0.47	13.69±1.26	48.47±1.07
FA2	0.85±0.06	0.81±0.01	1.59±0.01	4.36±0.08	45.33±2.49	182.74±4.81	10.32±2.28	0.17±0.01	0.11±0.00	0.23±0.02	6.55±0.26	-264.80±20.82	45.66±2.76	19.69±0.01	13.76±0.24	48.69±0.60
FA3	0.50±0.05	0.59±0.04	1.13±0.04	3.43±0.13	37.76±1.96	178.04±2.48	9.36±0.84	0.17±0.02	0.11±0.00	0.25±0.01	6.71±0.09	-246.55±16.75	61.97±2.68	20.03±0.14	14.29±0.27	47.02±0.15
FA4	0.63±0.05	0.54±0.03	1.04±0.11	3.02±1.09	38.57±1.44	154.91±7.16	8.64±1.44	0.16±0.02	0.11±0.01	0.22±0.01	6.59±0.13	-248.50±32.31	65.24±0.53	21.95±1.23	13.84±0.21	46.71±1.46
FA5	0.46±0.14	0.50±0.09	0.81±0.11	3.44±0.08	37.07±3.10	143.79±4.29	6.24±0.48	0.14±0.01	0.11±0.01	0.24±0.02	6.52±0.12	-260.53±34.43	138.91±18.72	22.08±0.01	14.73±0.20	45.54±0.41
FA6	0.50±0.02	0.51±0.06	0.88±0.03	3.44±0.16	35.89±2.37	116.60±1.13	6.08±0.73	0.14±0.00	0.11±0.00	0.24±0.01	6.35±0.20	-284.10±16.74	165.62±12.04	22.77±1.01	15.89±0.10	46.57±1.23
HA1	2.25±0.20	1.33±0.12	2.67±0.30	3.94±1.13	42.97±2.36	192.35±9.72	11.40±0.84	0.18±0.01	0.10±0.00	0.23±0.02	6.90±0.06	-222.63±53.46	22.78±1.02	20.36±0.15	13.44±1.27	48.92±0.44
HA2	1.44±0.09	0.78±0.01	1.41±0.15	4.06±0.53	39.64±2.18	192.19±4.73	9.30±0.18	0.15±0.01	0.10±0.01	0.21±0.01	6.95±0.06	-168.13±25.19	39.30±0.80	20.38±0.03	13.96±0.61	46.99±0.92
HA3	0.88±0.08	0.75±0.06	1.54±0.13	3.12±0.08	35.21±0.22	155.64±8.26	8.04±0.12	0.15±0.02	0.09±0.01	0.22±0.01	7.08±0.04	-244.40±21.60	63.69±1.45	21.34±0.16	13.13±0.55	47.94±0.98
HA4	0.69±0.08	0.69±0.03	0.88±0.01	3.73±0.11	35.22±1.31	138.83±6.98	7.44±0.36	0.12±0.01	0.09±0.01	0.22±0.01	7.19±0.09	-227.50±21.21	60.45±4.95	22.28±0.43	14.34±1.01	46.16±0.85
HA5	0.40±0.03	0.44±0.01	1.00±0.06	2.54±0.37	33.24±2.35	120.28±6.39	4.02±0.54	0.07±0.00	0.09±0.00	0.19±0.00	7.29±0.01	-252.13±41.37	91.27±3.83	23.97±0.88	14.08±0.69	47.15±0.12
HA6	0.25±0.05	0.49±0.01	0.90±0.01	3.09±0.63	31.33±2.25	118.94±9.80	4.14±0.54	0.07±0.00	0.09±0.00	0.20±0.00	7.37±0.06	-244.45±5.05	115.20±8.04	23.21±0.02	14.73±0.20	46.60±0.94

Table S4. Mean ± SE values of soil properties, extractable As, As fractions, plaque-associated metals, rice tissue As contents under different treatments. Data are means ± SE (*n* = 3).

	**StemAs**	**LeafAs**	**RootAs**	**AsIP**	**FeIP**	**MnIP**	**Available As**	**AEAs**	**AlAs**	**FeAs**	**DOC**	**SOC**	**pH**	**Eh**	**Feo**	**Fed**
CK	9.40±1.39	15.56±0.76	0.21±0.00	395.45±14.89	41.23±1.10	201.56±7.48	12.98±0.78	0.25±0.04	2.07±0.21	32.31±1.92	15.85±7.39	19.49±0.15	6.77±0.12	-171.15±25.45	12.21±0.08	49.79±0.54
FA1	10.54±0.68	16.76±1.59	0.19±0.03	402.46±18.98	39.77±2.18	155.61±3.67	14.37±0.90	0.38±0.03	3.66±0.21	26.14±2.28	40.92±1.09	19.96±0.47	6.73±0.07	-225.80±63.92	13.69±1.26	48.47±1.07
FA2	10.71±0.80	16.46±2.36	0.24±0.02	358.68±1.70	45.33±2.49	182.74±4.81	14.42±0.65	0.39±0.01	4.30±0.21	29.73±2.69	45.66±2.76	19.69±0.01	6.55±0.26	-264.80±20.82	13.76±0.24	48.69±0.60
FA3	9.93±0.00	14.80±2.00	0.25±0.04	358.79±12.72	37.76±1.96	178.04±2.48	16.03±1.55	0.40±0.04	3.73±0.30	23.19±0.61	61.97±2.68	20.03±0.14	6.71±0.09	-246.55±16.75	14.29±0.27	47.02±0.15
FA4	11.62±0.30	11.93±0.79	0.27±0.04	345.60±1.68	38.57±1.44	154.91±7.16	17.39±0.95	0.37±0.01	3.65±0.09	24.01±0.41	65.24±0.53	21.95±1.23	6.59±0.13	-248.50±32.31	13.84±0.21	46.71±1.46
FA5	8.15±0.70	11.48±0.59	0.32±0.05	340.47±9.50	37.07±3.10	143.79±4.29	18.76±2.36	0.41±0.02	3.67±0.16	24.89±2.54	138.91±18.72	22.08±0.01	6.52±0.12	-260.53±34.43	14.73±0.20	45.54±0.41
FA6	5.71±0.59	9.76±0.89	0.34±0.03	337.27±14.50	35.89±2.37	116.60±1.13	17.87±0.82	0.37±0.02	3.42±0.38	23.47±0.43	165.62±12.04	22.77±1.01	6.35±0.20	-284.10±16.74	15.89±0.10	46.57±1.23
HA1	8.20±0.99	11.57±0.88	0.30±0.02	361.23±17.98	42.97±2.36	192.35±9.72	14.35±2.04	0.28±0.02	3.49±0.10	24.70±1.16	22.78±1.02	20.36±0.15	6.90±0.06	-222.63±53.46	13.44±1.27	48.92±0.44
HA2	9.21±0.25	12.09±0.40	0.22±0.02	443.57±2.62	39.64±2.18	192.19±4.73	13.16±0.23	0.28±0.04	3.26±0.32	22.96±2.42	39.30±0.80	20.38±0.03	6.95±0.06	-168.13±25.19	13.96±0.61	46.99±0.92
HA3	11.05±1.66	13.72±0.28	0.25±0.02	518.08±10.08	35.21±0.22	155.64±8.26	15.96±0.90	0.31±0.02	3.91±0.22	23.96±1.32	63.69±1.45	21.34±0.16	7.08±0.04	-244.40±21.60	13.13±0.55	47.94±0.98
HA4	14.83±0.86	13.41±0.51	0.21±0.00	506.99±18.19	35.22±1.31	138.83±6.98	15.61±1.17	0.25±0.04	3.83±0.29	25.48±2.04	60.45±4.95	22.28±0.43	7.19±0.09	-227.50±21.21	14.34±1.01	46.16±0.85
HA5	15.87±0.19	14.24±0.87	0.20±0.02	550.52±9.78	33.24±2.35	120.28±6.39	15.03±0.65	0.29±0.07	3.58±0.04	33.21±0.99	91.27±3.83	23.97±0.88	7.29±0.01	-252.13±41.37	14.08±0.69	47.15±0.12
HA6	16.57±0.51	14.75±0.82	0.16±0.00	572.29±12.69	31.33±2.25	118.94±9.80	16.90±0.17	0.29±0.04	3.54±0.18	26.19±2.55	115.20±8.04	23.21±0.02	7.37±0.06	-244.45±5.05	14.73±0.20	46.60±0.94

Table S5. Effects of amendment type, carbon-equivalent dose, and their interaction on soil and rice variables.

Variable	Amendment type	Dose	Type × Dose
Grain Cd	0.204 ns	<0.001***	<0.001***
Grain As	<0.001***	0.001***	0.001***
pH	<0.001***	0.240 ns	<0.001***
Eh	0.017*	0.149 ns	0.160 ns
DOC	<0.001***	<0.001***	<0.001***
SOC	<0.001***	<0.001***	0.137 ns
Feo	0.079 ns	0.002**	0.214 ns
Fed	0.661 ns	<0.001***	0.045*
$CaCl_2$-Cd	0.004**	<0.001***	0.064 ns
EX-Cd	<0.001***	<0.001***	<0.001***
CA-Cd	<0.001***	0.237 ns	<0.001***
OX-Cd	<0.001***	0.081 ns	0.005**
OM-Cd	0.194 ns	0.215 ns	0.448 ns
RES-Cd	0.471 ns	0.253 ns	0.322 ns
KH_2PO_4-As	0.004**	<0.001***	0.129 ns
AE-As	<0.001***	0.295 ns	0.927 ns
Al-As	0.093 ns	0.123 ns	0.001***
Fe-As	0.179 ns	0.001***	<0.001***
Ca-As	0.211 ns	0.535 ns	0.613 ns
RES-As	0.274 ns	0.157 ns	0.384 ns
Fe_{IP}	0.001***	<0.001***	0.023*
Mn_{IP}	0.300 ns	<0.001***	<0.001***
Cd_{IP}	0.787 ns	0.031*	0.119 ns
As_{IP}	<0.001***	<0.001***	<0.001***
Stem Cd	<0.001***	<0.001***	<0.001***
Leaf Cd	<0.001***	<0.001***	<0.001***
Root Cd	<0.001***	<0.001***	<0.001***
Stem As	<0.001***	<0.001***	<0.001***
Leaf As	0.556 ns	0.013*	<0.001***
Root As	<0.001***	0.610 ns	<0.001***
$TF_{stem-grain}$ Cd	<0.001***	0.002**	0.033*
$TF_{stem-grain}$ As	<0.001***	<0.001***	<0.001***
$TF_{root-stem}$ Cd	0.146 ns	<0.001***	<0.001***
$TF_{root-stem}$ As	<0.001***	<0.001***	<0.001***

Note: Values are P values from the two-factor analysis of variance examining the effects of amendment type, carbon-equivalent dose, and their interaction. ns, $P > 0.05$; * $P \leq 0.05$; ** $P \leq 0.01$; *** $P \leq 0.001$.

Table S6. Dose-specific comparisons between FA and HA treatments under carbon-equivalent application rates.

**Variable**	**0.5 g C kg^{-1}**	**1 g C kg^{-1}**	**2 g C kg^{-1}**	**3 g C kg^{-1}**	**4 g C kg^{-1}**	**5 g C kg^{-1}**
Grain Cd	**−0.198 (<0.006)**	0.013 (1.000)	0.024 (1.000)	0.061 (0.186)	**0.090 (0.012)**	**0.096 (0.006)**
Grain As	0.011 (1.000)	−0.047 (0.150)	**−0.093 (<0.006)**	**−0.095 (<0.006)**	**−0.114 (<0.006)**	**−0.112 (<0.006)**
pH	−0.163 (0.654)	**−0.400 (<0.006)**	**−0.363 (0.006)**	**−0.600 (<0.006)**	**−0.770 (<0.006)**	**−1.020 (<0.006)**
DOC	**18.137 (0.030)**	6.361 (1.000)	−1.723 (1.000)	4.793 (1.000)	**47.642 (<0.006)**	**50.424 (<0.006)**
$CaCl_2$-Cd	−1.320 (0.684)	1.020 (1.000)	−1.320 (0.684)	1.200 (0.894)	2.220 (0.066)	1.940 (0.144)
KH_2PO_4-As	0.015 (1.000)	1.259 (1.000)	0.069 (1.000)	1.782 (0.504)	**3.726 (0.006)**	−0.975 (1.000)
Cd_{IP}	−0.830 (0.582)	0.298 (1.000)	0.311 (1.000)	−0.704 (0.936)	0.897 (0.444)	−0.350 (1.000)
As_{IP}	**41.231 (<0.006)**	**−84.893 (<0.006)**	**−159.290 (<0.006)**	**−161.390 (<0.006)**	**−210.054 (<0.006)**	**−235.018 (<0.006)**

Note: Values are mean differences between FA and HA treatments (FA − HA), with Bonferroni-adjusted *P* values in parentheses. Negative values indicate lower values under FA than under HA, whereas positive values indicate higher values under FA than under HA. *P* values were adjusted for the six dose-specific FA–HA comparisons within each variable using the Bonferroni method. Bold values indicate significant differences after adjustment (adjusted $P < 0.05$).

Table S7. Amendment-specific dose-response relationships for selected soil and grain variables under carbon-equivalent application rates.

Variable	Amendment	Selected model	R^2	Regression coefficient, B (95% CI)	P	Dose-response pattern
DOC	FA	Quadratic	0.927	β_1 = −5.382 (−25.524, 14.760); β_2 = 6.219 (2.598, 9.840)	β_1: 0.577; β_2: 0.002	Nonlinear increase
	HA	Linear	0.936	18.809 (16.210, 21.409)	<0.001	Linear increase
$CaCl_2$-Cd	FA	Linear	0.664	−1.030 (−1.419, −0.642)	<0.001	Linear decrease
	HA	Linear	0.907	−1.602 (−1.874, −1.330)	<0.001	Linear decrease
KH_2PO_4-As	FA	Linear	0.607	0.979 (0.561, 1.397)	<0.001	Linear increase
	HA	Linear	0.37	0.564 (0.174, 0.954)	0.007	Linear increase
Grain Cd	FA	Linear	0.788	−0.034 (−0.043, −0.025)	<0.001	Linear decrease
	HA	Linear	0.786	−0.084 (−0.107, −0.061)	<0.001	Linear decrease
Grain As	FA	Linear	0.583	−0.019 (−0.028, −0.011)	<0.001	Linear decrease
	HA	Quadratic	0.318	β_1 = 0.051 (0.008, 0.094); β_2 = −0.009 (−0.0175, −0.0005)	β_1: 0.020; β_2: 0.029	Curved response[1]

Note: Dose was treated as a continuous quantitative variable, and linear and quadratic regression models were fitted separately for FA and HA using the six nonzero carbon-equivalent application rates (0.5–5 g C kg^{-1} soil). Linear models were retained when the quadratic term was not significant. For quadratic models, β_1 and β_2 represent the linear and quadratic coefficients, respectively. Values in parentheses are 95% confidence intervals.

[1]For grain As under HA, the linear trend was not significant, and although the quadratic term was significant, the overall quadratic model was marginally non-significant (*P* = 0.057). Therefore, the response was interpreted cautiously as evidence of curvature rather than as a firmly established non-monotonic dose-response.
